# Cationic microparticles inhibit local sterile inflammation in tissue injuries

**DOI:** 10.1093/rb/rbaf135

**Published:** 2025-12-31

**Authors:** Ying Zhang, Hanyao Huang, Jaewoo Lee, Jin-Young Hong, Jong-Wan Kim, Hon Fai Chan, Jung-Keun Hyun, Bruce A Sullenger, Hae-Won Kim, Kam W Leong

**Affiliations:** Department of Biomedical Engineering, Columbia University, New York, NY 10027, USA; Department of Biomedical Engineering, Duke University, Durham, NC 27708, USA; Department of Biomedical Engineering, Columbia University, New York, NY 10027, USA; Department of Surgery, Duke University Medical Center, Durham, NC 27710, USA; Department of Nanobiomedical Science, Dankook University, Cheonan 31116, Republic of Korea; Institute of Tissue Regeneration Engineering, Dankook University, Cheonan 31116, Republic of Korea; Department of Biomedical Engineering, Columbia University, New York, NY 10027, USA; Department of Biomedical Engineering, Duke University, Durham, NC 27708, USA; Institute for Tissue Engineering and Regenerative Medicine, The Chinese University of Hong Kong, SAR, China; Department of Nanobiomedical Science, Dankook University, Cheonan 31116, Republic of Korea; Institute of Tissue Regeneration Engineering, Dankook University, Cheonan 31116, Republic of Korea; Mechanobiology Dental Medicine Research Center, Dankook University, Cheonan 31116, Republic of Korea; Department of Surgery, Duke University Medical Center, Durham, NC 27710, USA; Department of Nanobiomedical Science, Dankook University, Cheonan 31116, Republic of Korea; Institute of Tissue Regeneration Engineering, Dankook University, Cheonan 31116, Republic of Korea; Mechanobiology Dental Medicine Research Center, Dankook University, Cheonan 31116, Republic of Korea; Department of Biomedical Engineering, Columbia University, New York, NY 10027, USA; Department of Systems Biology, Columbia University Medical Center, New York, NY 10032, USA

**Keywords:** toll-like receptor, sterile inflammation, polycations, microparticles, microfluidics

## Abstract

Sterile inflammation driven by circulating cell-free nucleic acids (cfNAs) plays a critical role in immune activation across various pathological conditions, including autoimmune diseases and tissue injuries. In this study, we explore the potential of polycation-coated microparticles as localized cfNA scavengers designed to mitigate inflammation without the risk of cellular uptake that could reactivate toll-like receptor (TLR) pathways. Using chemical-induced cell death models, we show that the amount of cfNAs released varies depending on cell type and drug treatment. The cationic microparticles effectively inhibit TLR activation by various cfNA forms, including ssDNA, dsRNA, DNA–antibody complexes, as well as cfNAs derived from dying-cell supernatants and lupus patient plasma. Microparticles coated with polycations such as protamine sulfate and poly-L-lysine blocked cfNA uptake and reduced TLR activation by 40–60% in cell culture models. *In vivo*, they demonstrated therapeutic efficacy by mitigating acute peritonitis, reducing inflammation in temporomandibular joint (TMJ) dysfunction, and minimizing secondary tissue damage in spinal cord injury. Additionally, we used a microfluidics-based approach to fabricate uniform, biocompatible chitosan microparticles with enhanced cfNA-scavenging capabilities. These findings highlight the potential of polycation-coated microparticles as a localized strategy for mitigating cfNA-driven inflammation and promoting tissue recovery in sterile inflammatory diseases.

## Graphical abstract

**Figure:**
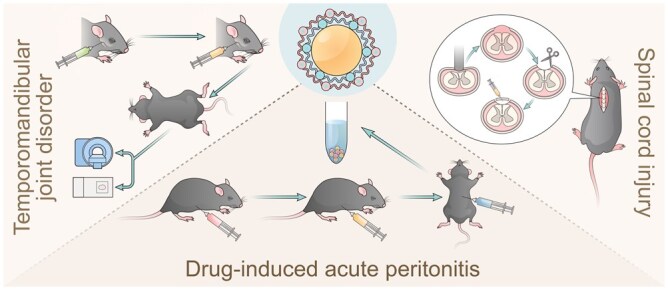

## Introduction

Sterile tissue injury, similar to microbial infections, provokes strong immune responses that compromise tissue integrity and exacerbate damage [1]. However, unlike the immune response to microbial infections driven by pathogen-associated molecular patterns (PAMPs) from foreign organisms, tissue injury-induced immune responses are triggered by endogenous damage-associated molecular patterns (DAMPs) [2, 3]. A part of major DAMPs involved in activating innate immune responses are cell-free nucleic acids (cfNAs), which originate from genomic DNA, mRNA, ribosomal RNA or mitochondrial DNA, either as free molecules or in protein-bound states [4]. During massive cell death triggered by tissue injury, surgery, transplantation, or chemotherapy, pathogenic nucleic acids (NAs) are released and sensed by toll-like receptors (TLRs) in the innate immune system—for example, DNA activates TLR9—thereby inducing robust inflammatory responses characterized by elevated levels of cytokines such as TNF-α and IL-6 [5–11]. Evidence demonstrates that elevated levels of cfNAs in local tissues or systemic circulation are associated with various pathological sterile disorders, including muscle fibrosis, osteoarthritis, temporomandibular joint disorder (TMD), and systemic lupus erythematosus (SLE) [5, 12–14].

Since NAs are negatively charged, the cationic cfNA-scavenging strategy leverages positive–negative charge interactions to capture cfNAs, thereby inhibiting cfNA-induced inflammatory responses [15]. We have previously shown that this approach is effective in treating a range of inflammatory diseases through various formulations. Cationic nanoparticles have shown versatility by scavenging cfNAs, inhibiting TLR activation, delivering drugs, and enhancing tissue penetration in conditions such as rheumatoid arthritis, sepsis, inflammatory bowel disease, chronic obstructive pulmonary disease, periodontitis, muscle fibrosis and cancer therapy [5, 16–21]. Similarly, cationic hydrogels have effectively cleared cfNAs while releasing anti-inflammatory agents like IL-10, and exhibiting antibacterial and self-healing properties in applications such as spinal cord injury, periodontitis, and severe abdominal trauma [22–24]. Overall, the cationic cfNA-scavenging strategy presents a promising and effective approach for inflammation treatment.

In sterile tissue injury, pathogenic NAs released from damaged tissue need to re-enter cells to activate intracellular NA-recognizing TLRs [25–27]. Therefore, both scavenging cfNAs and preventing their cellular entry are crucial considerations. Current nanoparticulate cfNA scavengers, due to their small size, can easily enter cells, prompting the search for a more effective alternative[5, 16, 17]. A microparticle-based approach, which has not been previously reported, offers advantages such as a large size to limit cellular entry and extensive surface area for complexation with larger ligands. Given the intracellular localization of NA-recognizing TLRs, this study introduces immobilized cationic microparticles as cfNA scavengers. We develop these cationic microparticles and assess their potential as broad-spectrum anti-inflammatory agents in three animal models: drug-induced acute peritonitis, spinal cord injury and TMD (Supplementary Figure S1).

## Results

### Cell death, cfNAs and inflammation

**Figure 1:**
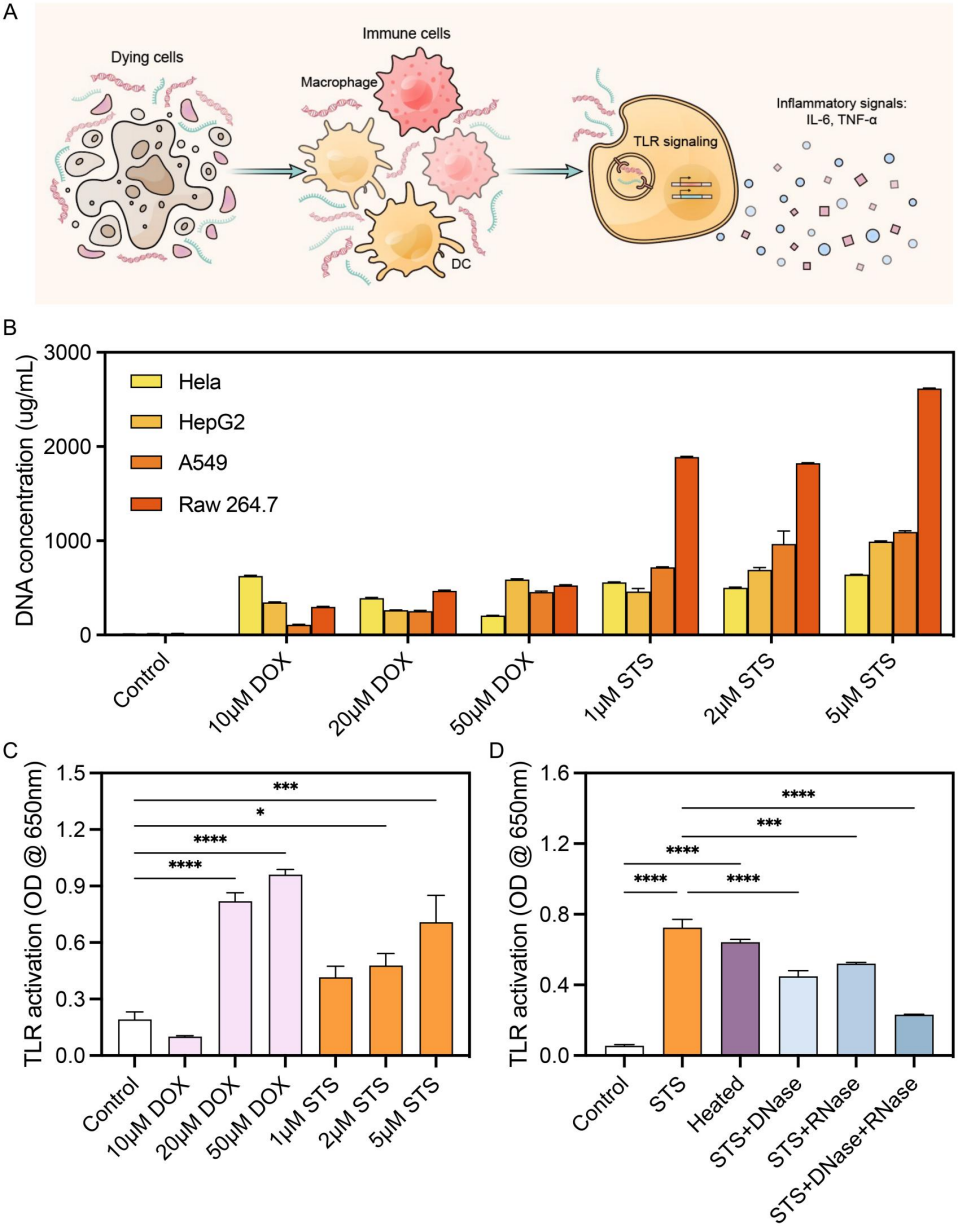
cfNAs from dead or dying cells induce sterile inflammation. (**A**) Schematic illustration of NA-induced sterile inflammation caused by cell death. (**B**) Quantification of DNA released by cell lines undergoing chemical-induced cell death. (**C**) Supernatant harvested from dead or dying cells activated TLR response in Ramos-blue™ reporter cells. (**D**) TLR activation by supernatant from dying cells was inhibited by DNase and RNase (*P < 0.05, ***P < 0.001, ****P < 0.0001; *n* = 6, error bar is standard deviation).

cfNAs released from dead or dying cells can activate TLR in immune cells, such as macrophages and dendritic cells, which release pro-inflammatory cytokines such as TNF-α and IL-6 (Figure 1A). To investigate the relationship between cell death, cfNAs release and inflammation activation, HeLa (human cervix adenocarcinoma cell line), HepG2 (human hepatocellular carcinoma cell line), A549 (human lung carcinoma cell line) and RAW 264.7 cells were treated with different concentrations of apoptosis inducers, staurosporine (STS) or doxorubicin (Dox). The amount of cfNAs (represented by cfDNA) released upon cell death varied with drug treatment and cell type (Figure 1B). STS-treated cells released more cfNA fragments as compared with Dox-treated cells. RAW 264.7 cells, as the macrophage cell line, released more NAs than other cell lines when treated with STS.

To assess whether the released molecules would activate the immune response, we harvested the supernatant from drug-treated cells and supplemented it to the medium of Ramos-Blue™ cell culture at a volume ratio of 1:5. Both Dox- and STS-treated cell supernatant induced strong TLR activation (Figure 1C), and the amplitude of TLR activation depended on drug concentration. When DNase and RNase were added to the dying-cell supernatant, TLR induction was significantly suppressed (Figure 1D), suggesting that cfNAs acted as the activators.

### Cationic microparticle preparation and inhibition of cfNA-induced TLR activation

**Figure 2:**
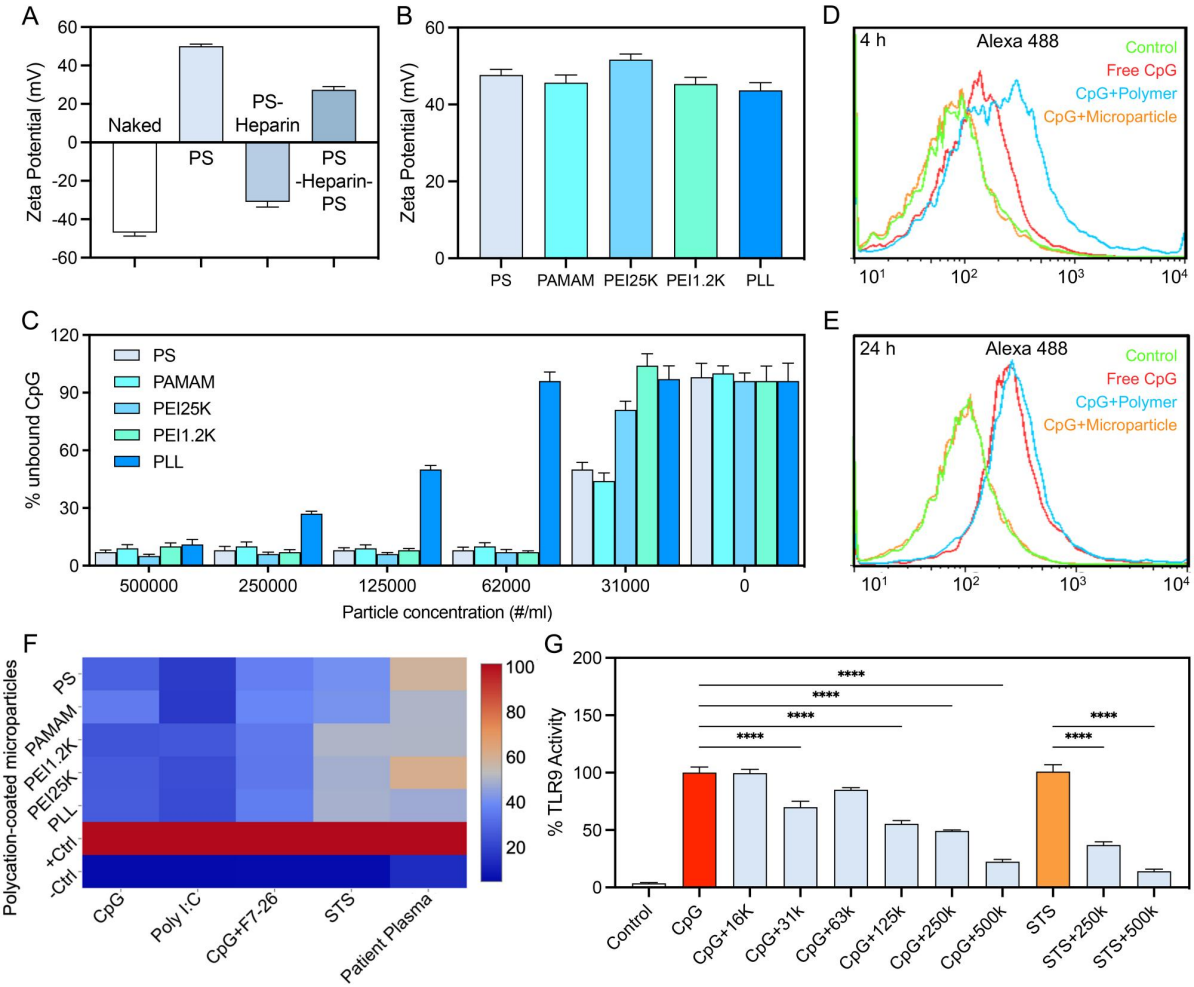
Cationic polystyrene microparticles function as cfNA scavengers. (**A**) Stepwise changes in Zeta potential of carboxylate polystyrene microparticles after layer-by-layer coating with PS and heparin. (**B**) Final Zeta potential of microparticles coated with different polycations. (**C**) The binding efficiency of polycation-coated microparticles toward CpG is concentration-dependent. (**D, E**) CpG uptake by dendritic cells (DC2.4) 4 h (**D**) and 24 h (**E**) after incubation with PS-coated microparticle. (**F**) Heatmap of TLR activation induced by various NA-containing agonists with or without polycation-coated microparticles (0.5 × 10^6^ microparticles/mL). Agonist concentration used was: CpG (1 μM), poly(I:C): C (10 μg/mL), CpG anti-DNA immune complex (1 μM CpG + 0.6 μg/mL F7–26), supernatant from dying cells (STS sup) (40% v/v), lupus patient plasma (10% v/v). (**G**) Dose-dependent inhibition of TLR9 activity by polycation-coated microparticles (****P < 0.0001; *n* = 6, error bar is standard deviation).

To assess whether microparticles coated with polycations could function as effective cfNA scavengers, we first used commercial polystyrene beads for proof-of-concept because they are both biologically and immunologically inert [28]. Polystyrene beads containing carboxyl groups with a surface charge of −50 mV were used (Figure 2A). When coated with protamine sulfate (PS), the surface charge changed to +50 mV. Further coating with a negatively charged polymer, heparin, brought the surface charge to negative again (−32 mV), and another layer of coating with polycation reverted the surface charge to positive (+20 mV) (Figure 2A). This initial layer-by-layer coating experiment demonstrated the robustness of the protocol and the evolution of the surface charge. Subsequent experiments were all done with only one coating.

Five commonly used polycations were selected to coat the microparticles, including PS, 1,4-diaminobutane-poly (amino-amine)-G3 (PAMAM), 25 kDa branched polyethyleneimine (PEI25K), 1.2 kDa polyethyleneimine (PEI1.2K), and poly-L-Lysine (PLL). Despite their apparent difference in DNA binding affinity [29, 30], they led to a similar Zeta potential of the polystyrene microparticles when immobilized on the microparticle surface (Figure 2B). Coating with a higher polymer (PS) concentration did not produce a higher Zeta potential (Supplementary Figure S2). Microparticles coated with different polycations could bind CpG efficiently in a concentration-dependent manner (Figure 2C), except for PLL, which appeared to be the weakest. Meanwhile, adsorption of CpG by coated microparticles prevented their cellular uptake (Figure 2D and E).

Next, we examined whether the cationic microparticles could inhibit TLR activation in the Ramos-Blue™ reporter cells. The results were plotted in a heatmap in Figure 2F for various TLR agonists and polycation-coated microparticles. Cationic microparticles demonstrated the ability to inhibit TLR9 activation by CpG and poly(I:C) with an efficiency of nearly 90%. In most pathological conditions, cfNAs are often bound by anti-DNA antibodies, nuclear proteins or other cationic proteins [31, 32]. Complexing CpG with an ssDNA binding antibody F7-26 resulted in higher TLR activation as compared to free CpG alone (Supplementary Figure S3). Cationic microparticles could reduce the CpG-F7-26-induced TLR activation by 70% (Figure 2F). We also tested whether cationic microparticles were effective against endogenous NA-induced TLR activation, such as those from dying cells and from lupus patient plasma. Cationic microparticles, depending on the coating polycations, achieved a 40% to 60% inhibition toward TLR activation by dying-cell supernatant. In addition, their inhibition effect against lupus patient plasma was about 50% to 60% depending on the coating polycations (Figure 2F). The decrease in potency against those endogenous agonists was expected, as they usually comprised more complex structures such as NA-protein complexes and NA-containing vesicles. Meanwhile, the inhibition of TLR activity by coated microparticles was dose-dependent (Figure 2G).

### Attenuation of STS-induced acute peritonitis

**Figure 3:**
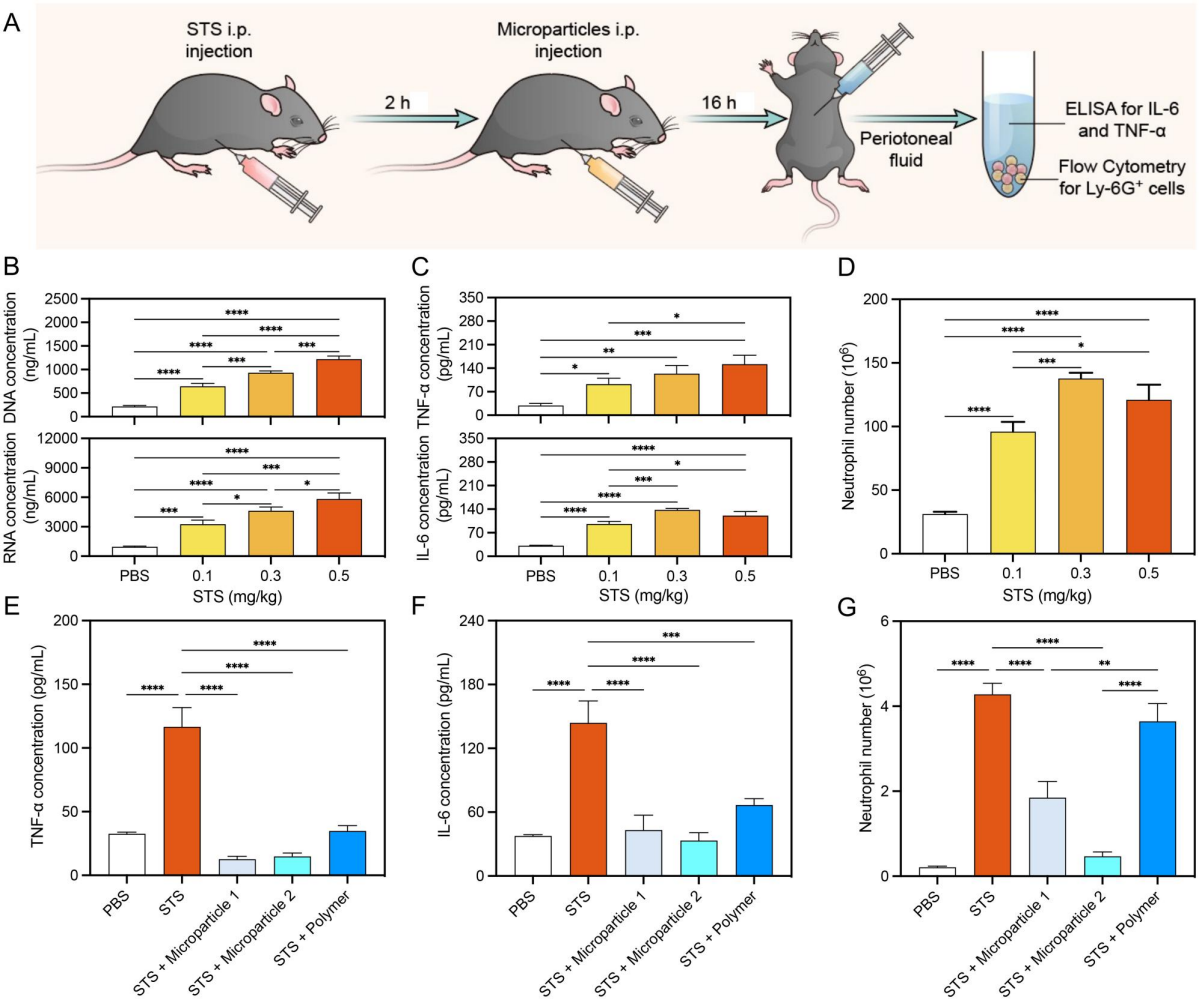
PS-coated microparticles suppressed the inflammatory response in STS-induced acute peritonitis. (**A**) Schematic illustration of STS-induced acute peritonitis animal study. Intraperitoneal administration of STS-induced acute inflammation as evidenced by the elevated (**B**) cfNAs concentration, (**C**) pro-inflammatory cytokine concentration, and (**D**) neutrophil recruitment in the peritoneal cavity. Administration of PS-coated microparticles 2 h (STS + microparticle 1) or both 2 h and 6 h (STS + microparticle 2) post-STS injection significantly lowered the cytokine level for both (**E**) TNF-α and (**F**) IL-6. (**G**) Neutrophil recruitment to the inflammatory site after microparticle administration (*P < 0.05, **P < 0.01, ***P < 0.001, ****P < 0.0001; *n* = 6, error bar is standard deviation).

Although the exact etiology of many inflammatory and autoimmune diseases is not fully understood, they are characterized by a highly destructive tissue environment associated with high levels of DAMP accumulation. To simulate such a destructive environment *in vivo*, acute peritonitis was first chosen and induced by intraperitoneal injection of STS (Figure 3A). STS is a potent apoptosis inducer that often results in the release of excessive DNA fragments after addition to cell cultures *in vitro* [33] and systemic administration *in vivo* [34]. Administration of STS resulted in an acute inflammatory response accompanied by increased cfNA level, increased IL-6 and TNF-α levels, and an influx of neutrophils, and the severity of inflammation correlated with the dosage of STS (Figure 3B–D). This observation was consistent with previous studies, which showed that intraperitoneal injection of Dox induced a severe inflammatory response [35].

Both TLR9 knockout and administration of a TLR antagonist reduced the Dox-induced inflammation, suggesting the critical role of TLR9 in the cfNA-mediated inflammatory response [35, 36]. Thus, we tested whether cationic microparticles could act as a TLR antagonist in STS-induced acute peritonitis. Free PS (STS + Polymer group), or PBS solution, was intraperitoneally injected 2 h after STS injection. PS-coated microparticles were used as single- or multi-doses, including 2 h after STS injection (STS + Microparticle 1), and 2 and 6 h after STS injection (STS + Microparticle 2). Peritoneal lavage was collected 16 h after STS administration and analyzed for the presence of neutrophils and inflammatory cytokines. Both microparticles and free polymer showed inhibition of inflammation in the peritoneal space, as demonstrated by the reduced level of cytokines (Figure 3E and F) and neutrophils (Figure 3G). Cationic microparticles were more effective toward neutrophil recruitment as compared with free polymer. These results suggested that cationic microparticles could play a protective role in STS-induced tissue damage and inflammation.

### Promotion of tissue recovery in TMJ dysfunction symptoms

**Figure 4:**
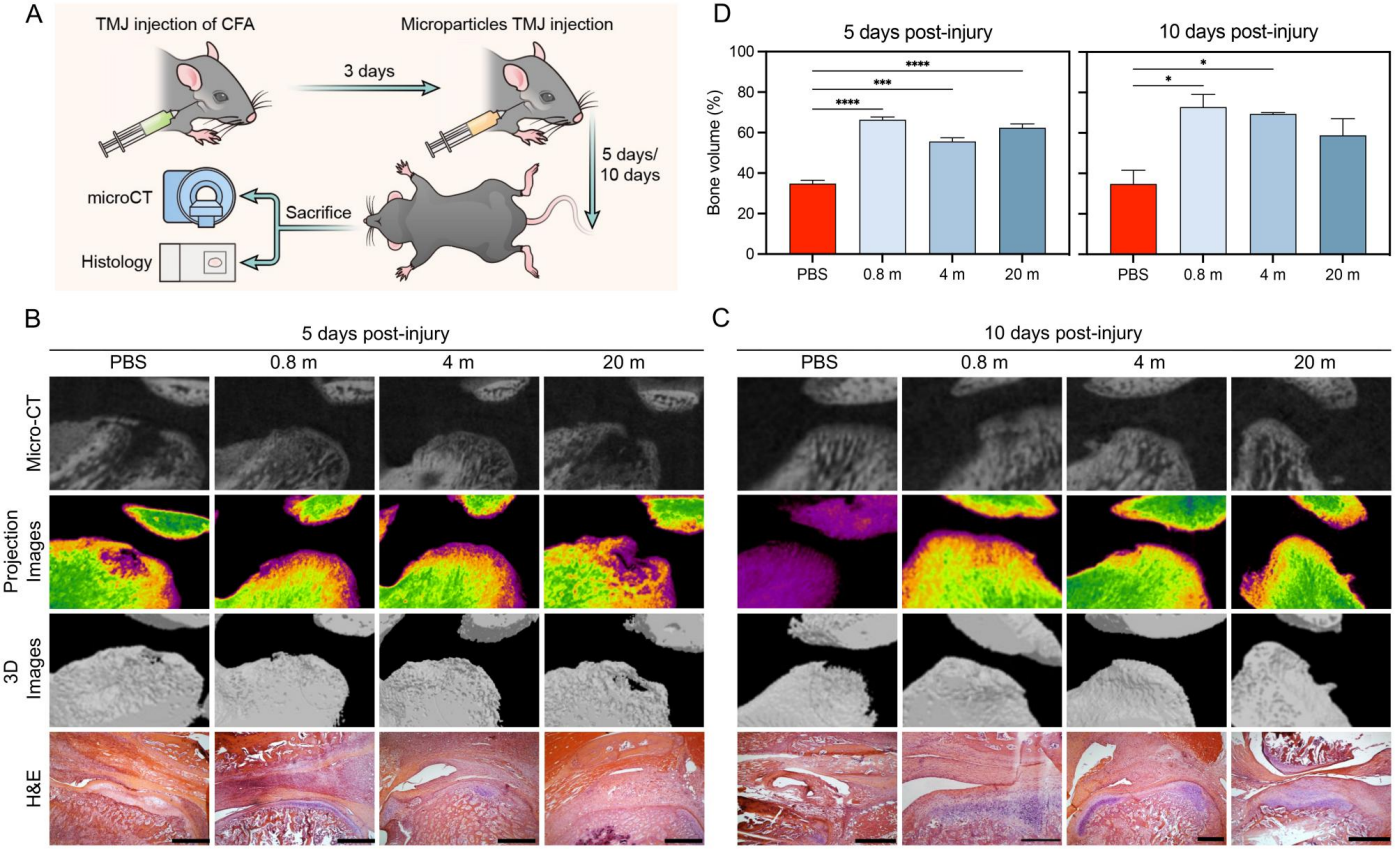
PS-coated microparticles promoted cartilage deposition and bone recovery in TMD. (**A**) Schematic illustration of the TMD animal study. (**B, C**) Micro-CT, projection images, 3D images, and H&E staining of the TMJ in mice showing the cartilage lining of the joint at day 5 and day 10 after treatment. Scalebar is 200 µm. (**D**) Bone volume in injured TMJ treated with various concentrations of cationic microparticles evaluated by micro-CT imaging of the joint. m, 1 × 10^6^ microparticles (*P < 0.05, ***P < 0.001, ****P < 0.0001; *n* = 3, error bar is standard deviation).

Next, we examined whether the cationic microparticles displayed protective function in disease conditions involving severe tissue damage. A TMJ dysfunction mouse model, which involves persistent inflammation at the TMJ and degradation of the cartilage lining and bone, was used for this study [37]. Intra-articular injection of complete Freund’s adjuvant (CFA) was employed to induce TMJ inflammation (Figure 4A and Supplementary Figure S4). Intra-articular administration of 0.8 million (m) PS-coated polystyrene microparticles helped restore the proper cartilage lining 5 and 10 days post-injury (Figure 4B and C). However, increasing the dose to 4 m and 20 m reduced the efficiency, suggesting a higher dose of microparticles was not desirable (Figure 4B and C). The microparticles also promoted bone formation, as evidenced by the increased bone volume in the jaw joint (Figure 4D). These results together suggest a positive role of the cationic microparticles in resolving local inflammation and promoting tissue recovery.

### Protective role in spinal cord injury in mice

**Figure 5:**
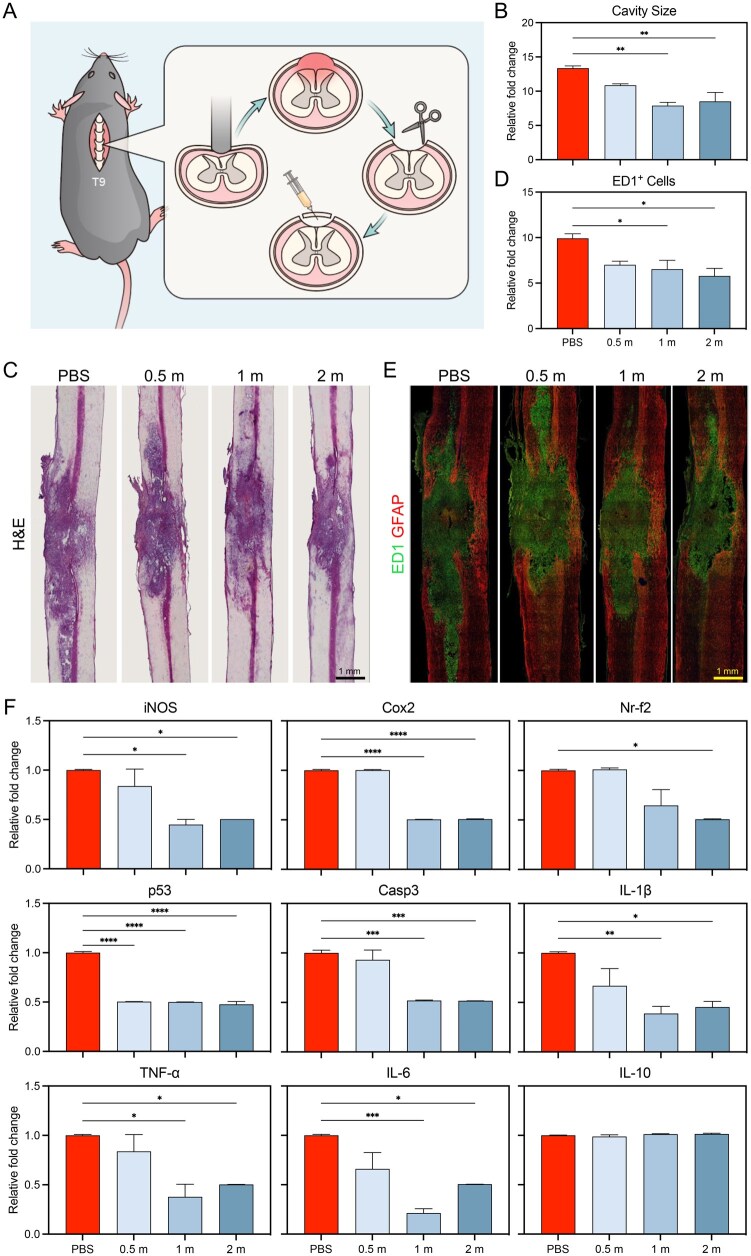
PS-coated microparticles reduced inflammation at the spinal cord injury site. (**A**) Schematic illustration of the spinal cord injury animal study. (**B**) Relative fold change in the cavity size of the injured site. (**C**) H&E staining of the injured spinal cord after treatment with different doses of PS-coated microparticles. (**D**) Relative fold change of the number of ED1^+^ cells in the injured site and (**E**) immunofluorescence staining of the ED1^+^ macrophage cells in the injured spinal cord after treatment with different doses of coated microparticles. (**F**) Relative changes of gene expression after treatment with different doses of coated microparticles in the spinal cord injury model. m, 1 × 10^6^ microparticles (*P < 0.05, ***P < 0.001, ****P < 0.0001; *n* = 3, error bar is standard deviation).

We also examined whether the cationic microparticles could provide a similar protective function against sterile inflammation in another destructive tissue condition. Spinal cord injury is characterized by prolonged inflammation [38]. The recruitment of immune cells to the injured tissue causes secondary injuries, which exacerbate the condition [39]. In this study, cationic microparticles were administered to the lesion site after the mice received a moderate contusion injury in the T9 spinal cord (Figure 5A). The lesions were reduced in all experimental groups compared with the control and accompanied by a reduction in cavity size (Figure 5B and C). Immunofluorescence staining of the ED1^+^ macrophage cells revealed an influx of the immune cells to the lesion, and treatment with cationic microparticles reduced the macrophage infiltration by 20% (Figure 5D and E). Cationic microparticles alleviated oxidative stress, apoptosis and inflammation in damaged tissue by reducing related gene expression (Figure 5F).

### Microfluidics-aided production of chitosan-based cationic microparticles with NA scavenging properties

After demonstrating the scavenging approach with polystyrene beads, which are not biodegradable, we tried to design microparticles with a more biocompatible biomaterial. Chitosan is a biocompatible polysaccharide that has been widely used for different pharmaceutical applications [40]. It degrades slowly to non-toxic amino sugars, which the body can completely absorb. Currently, the most common method for making chitosan microparticles is spray drying [41]. However, it produces a heterogeneous population of microparticles with a wide distribution of sizes. We developed a new method for preparing well-controlled and homogenous chitosan microparticles.

**Figure 6:**
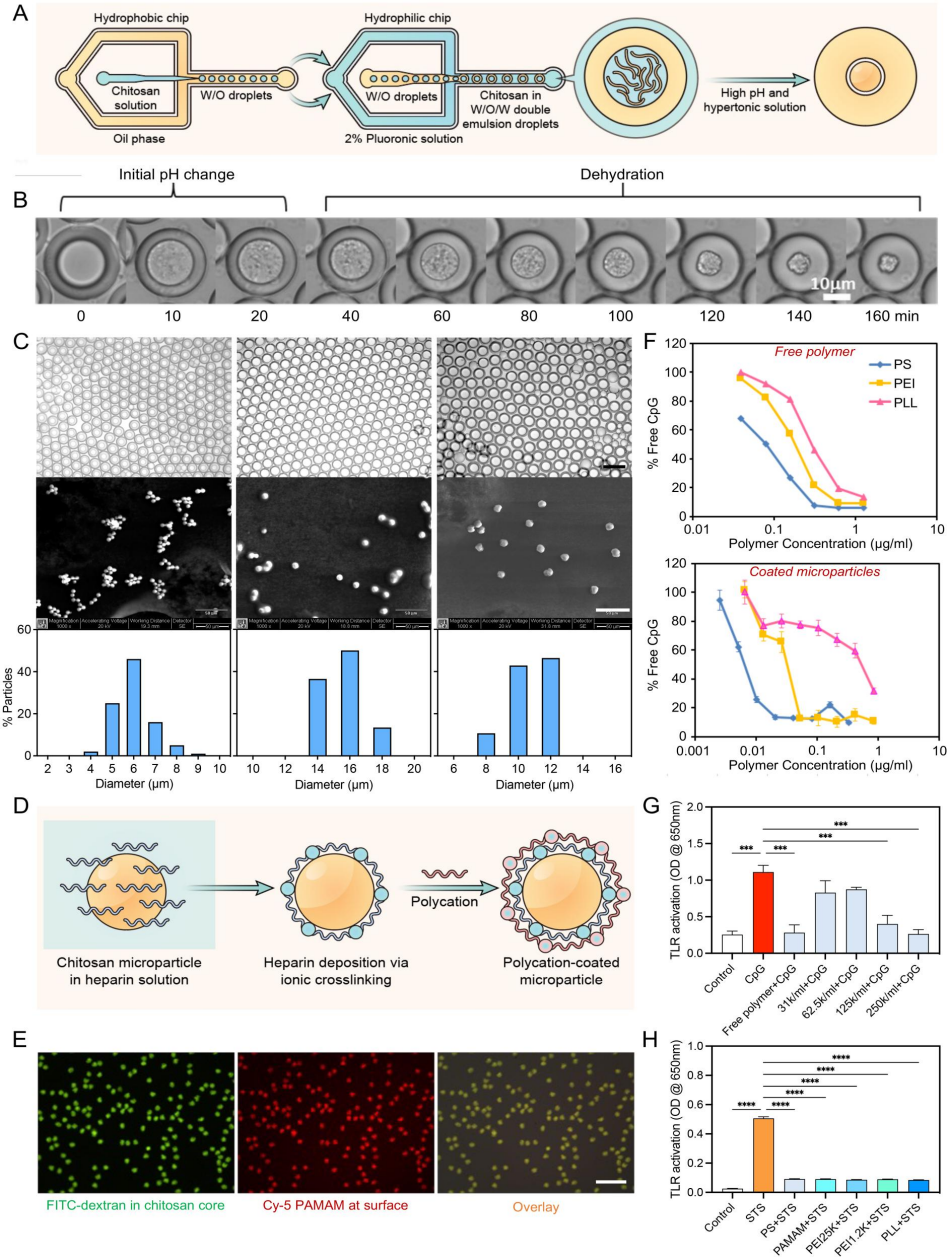
Fabrication and properties of chitosan-based cationic microparticles. (**A**) Schematic illustration of the setup, comprising two microfluidic chips connected in series to generate uniform W/O/W DE containing chitosan dissolved in pH 5.2. (**B**) When immersed in a pH 10 buffer containing 10% glycine, the chitosan solution was dehydrated to form a microgel particle over a period of 3 h. (**C**) Formation of chitosan microparticles with different sizes controlled by the microfluidics-generated DE. Top panel: microscope image of double emulsions collected from microfluidics chips (scalebar is 200 µm); Middle panel: SEM images of chitosan microparticles collected (scalebar is 50 µm); bottom panel: size distribution of chitosan microparticles (obtained by analysis of SEM images by ImageJ). (**D**) Schematic illustration for coating of the microparticles with polycations via an initial deposition of heparin followed by adsorption of polycations. (**E**) The fluorescence images of microparticles with uniform polycation coating and particle size. Scalebar is 50 µm. (**F**) Comparison of CpG binding affinity of free polycations and polycation-coated microparticles. (**G**) TLR activation by CpG. (**H**) TLR activation by supernatant from dying cells (***P < 0.001, ****P < 0.0001; *n* = 6, error bar is standard deviation).

Chitosan-based microparticles were prepared via a well-controlled gelation mechanism from microfluidics-generated double emulsions. Two microfluidic chips were connected in series to generate uniform water-in-oil-in-water (W/O/W) double emulsion droplets containing chitosan dissolved in a pH 5.2 sodium acetate buffer (Figure 6A). Biocompatible fluorocarbon oil was used as the oil phase and served as a barrier to prevent the out-diffusion of chitosan, while still allowing the movement of small ions and water. The primary amino group in the side chain of chitosan has a pKa of around 6.5, which protonates in acidic solutions. At neutral and alkaline pH, deprotonation of the amino groups renders chitosan hydrophobic with decreased solubility in water. After droplet collection, the double emulsions were transferred to a solution with high pH and high salt concentration (10% w/v glycine, pH 10). The chitosan inside the double emulsion droplets gelled within 20 min (Figure 6B). The high osmotic pressure created by 10% w/v glycine drove water to flow out of the core. Within 3 h, the chitosan in the core was dehydrated to form condensed microgel particles. The size of the particles could be controlled by the microfluidic channel dimensions and the flow rate ratio of the oil and aqueous phases [42]. We demonstrated the production of chitosan microparticles with an average diameter of 6, 10, and 16 μm and a narrow size distribution, as shown by the SEM images (Figure 6C). After breaking off the droplets and washing in PBS, the chitosan microparticles were coated with heparin, based on ionic crosslinking. The microparticles were then further coated with polycations through electrostatic interactions (Figure 6D). For imaging purposes, fluorescein isothiocyanate-labeled (FITC-labeled) dextran was incorporated into the chitosan core. Cy5-labeled PAMAM was used to coat the outer layer. Co-localization of these two dyes on the microparticles confirmed the layer-by-layer coating (Figure 6E).

The NA binding affinity of the polycation-coated chitosan microparticles was evaluated for their capacity to capture CpG 1668 (0.3 μM). The immobilized polycations were more effective than the free polycation counterparts in adsorbing the same amount of NA (Figure 6F). PS was the most effective among the three polycations tested. These data support our hypothesis that the immobilization of polycations on a microparticle surface enhanced their binding affinity toward NA due to a high local charge density and cooperative binding. Adsorption of CpG by polycation-coated chitosan microparticles also prevented their cellular uptake (Supplementary Figure S5). The PS-coated chitosan microparticles could inhibit CpG-induced TLR activation in Ramos-Blue™ reporter cells in a dose-dependent manner, and the potency was comparable to free polycation-mediated TLR inhibition (Figure 6G). We also showed that these cationic chitosan microparticles could inhibit dying-cell supernatant-induced TLR activation (Figure 6H). The microparticles were not cytotoxic at a concentration of at least up to 200 μg/mL (corresponding to 500K microparticles/mL) as evaluated by an MTT assay in RAW264.7 cells (Supplementary Figure S6).

### Comparison of cationic polystyrene microparticles and cationic chitosan microparticles in the TMJ dysfunction model

**Figure 7:**
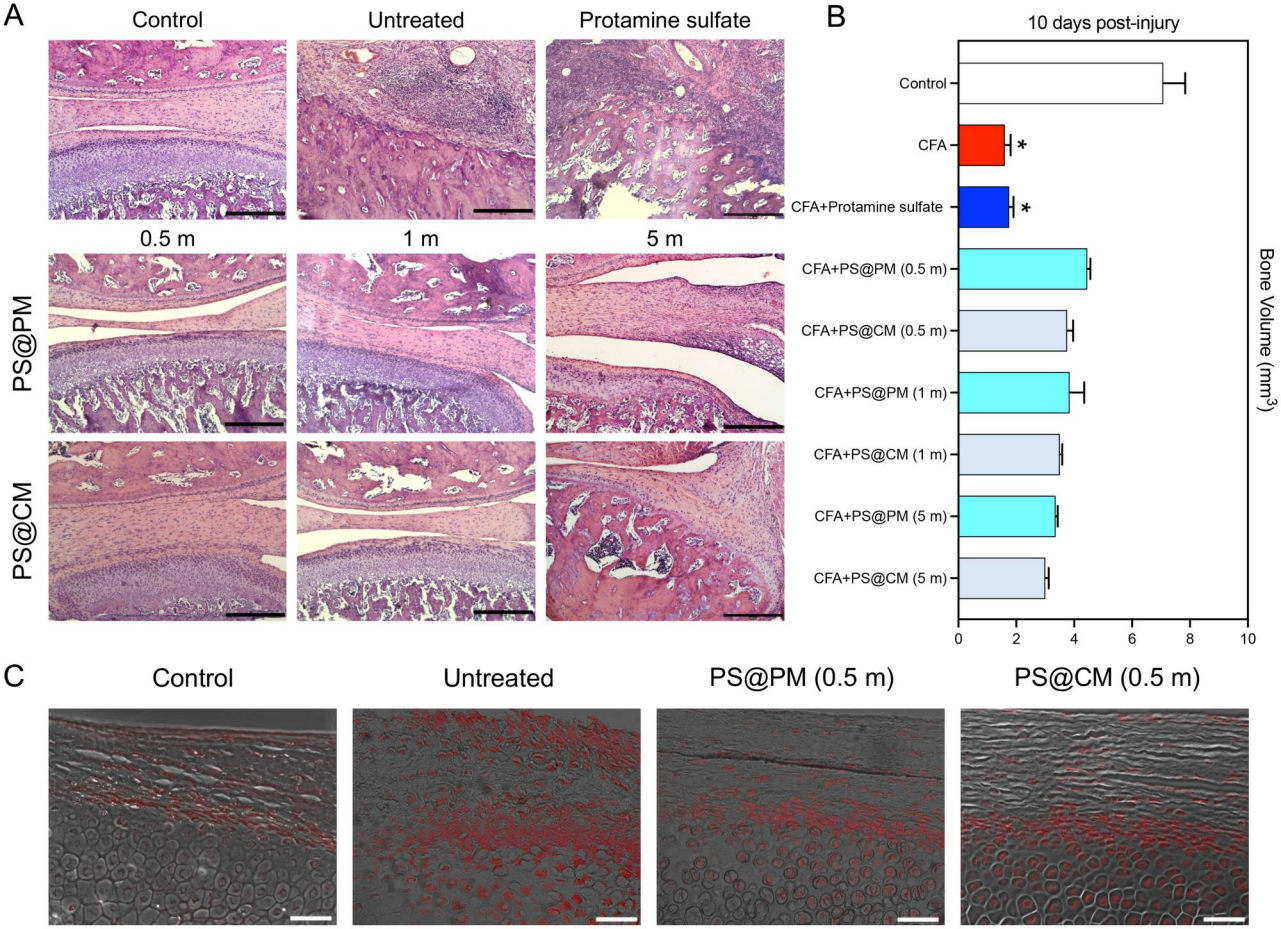
Comparison of cationic polystyrene microparticles and chitosan microparticles in the TMD model. (**A**) H&E staining of the TMJ in mice showing the cartilage lining of the joint at day 10 after different treatments. Scale bar is 200 µm. (**B**) Bone volume in injured TMJ treated with various concentrations of cationic microparticles evaluated by Micro-CT imaging of the joint. (**C**) DNA staining of the TMJ after two cationic microparticle treatments. PS@PM, PS-coated polystyrene microparticles; PS@CM, PS-coated chitosan microparticles. m, 1 × 10^6^ microparticles. Scalebar is 50 µm (* means the CFA group and CFA+PS group were significantly different from the other seven groups; *n* = 6, error bar is standard deviation).

We compared the potency of PS-coated chitosan microparticles (PS@CM) with PS-coated polystyrene microparticles (PS@PM) in the TMJ dysfunction model. Microparticles were administered at a lower dosage as suggested by the earlier study. Histological staining revealed that 1 m polystyrene microparticles and 0.5 m of chitosan microparticles could almost completely restore the cartilage lining of the injured TMJ (Figure 7A). Once again, a higher dosage of the microparticles showed a reduced effect. The bone volume was comparable for mice treated with polystyrene and chitosan microparticles (Figure 7B), and both showed significant improvement over the untreated groups. Interestingly, free PS, the polymer used to coat both microparticles, showed no effect on the cartilage lining or bone volume. We also stained the NA present in the damaged site using a cellular probe that stains NA from dead or dying cells. Due to the presence of intracellular NA, fluorescence staining did not reveal the presence of extracellular NA (Figure 7C). However, samples from mice receiving microparticle treatment showed fewer dead cells near the tissue surface, again indicating the protective role of these microparticles in the inflamed tissue.

## Discussion

Current clinically approved drugs to be used in sterile inflammation conditions, such as methylprednisolone, have ambiguous benefits and debilitating side effects. These drugs are typically administered systemically at high doses, which are often associated with non-specific tissue injury and paradoxically increased inflammation [43]. As cfNA recognition by the innate immune system is a significant cause of sterile inflammatory responses and accounts for several auto-inflammatory disorders, cfNA-sensing TLRs have become attractive therapeutic targets for the treatment of patients with these devastating inflammatory disorders [3]. cfNA-based antagonists have also been used as a strategy to prevent unwanted TLR activation [44–46]. However, this is unlikely to be optimal because of the interconnectedness and redundancy of TLR signaling. No matter which TLR is activated, TLR signaling culminates in the activation of MAP kinases, NF-κB, and IFN regulatory factors to elicit inflammatory cytokines. Moreover, inhibition of TLR signaling is deleterious for the innate immune defense of the body.

We presented a fundamentally different approach to simultaneously inhibit the inappropriate activation of multiple cfNA-sensing receptors for maximal effect. This was made possible by the fact that cationic polymers can bind NAs in a sequence-independent manner [15]. We have demonstrated that cationic polymers act as anti-inflammatory agents by scavenging NA to inhibit thrombosis without increasing bleeding [30]. This concept was expanded with polycationic nano- and micro-fibers [47, 48], followed by the development of cationic nanoparticles for rheumatoid arthritis by targeting cfNA and inhibiting TLR9 activation [16]. Similar strategies were applied to sepsis, inflammatory bowel disease, osteoarthritis, and refractory muscle fibrosis, where multifunctional nanoparticles scavenged cfNA and ROS [5, 13, 17, 18]. Dual-functional hydrogels were designed to clear DAMPs and release IL-10 for spinal cord injury [22]. Innovations such as cationic drug-delivering nanoparticles were used to inhibit chemotherapy-induced breast cancer metastasis and reduce obesity-related inflammation [21, 49, 50]. For periodontitis, cationic hydrogels and selenium-doped nanoparticles promoted bone regeneration while mitigating inflammation [20, 23]. In severe abdominal trauma, self-healing antibiotic hydrogels and polyaminoglycosides demonstrated anti-inflammatory and antibacterial effects [24]. Most recently, pH-responsive cationic nanoparticles enhanced mucus penetration for chronic obstructive pulmonary disease [19]. These studies highlight our evolving strategies to target cfNA-induced inflammatory pathways while integrating multifunctional and clinically relevant solutions [15]. However, the aforementioned studies found that free polycations can form nanocomplexes with cfNA *in situ*, facilitating re-entry into cells at the site. Thus, this work differs conceptually from these previous studies by demonstrating that immobilizing polycations on the microparticle surface prevents cellular NA entry and significantly suppresses NA-induced inflammatory responses, proposing the use of immobilized polycations for safer and more effective NA scavenging.

Cationic microparticles and free polycations function as NA scavengers via different mechanisms. Cationic microparticles bind with NA and could completely inhibit their cellular entry because of their size, which may minimize the encounter of extracellular cfNAs and intracellular TLRs. Free polycations, in contrast, facilitate the cellular entry of cfNAs [51]. After being internalized, they prevent TLR activation by competing for NA-binding. This explains why free polycations with relatively weak NA-binding affinity, such as PS and PLL, are not effective against cfNA-induced inflammation in our previous study [29] but function as efficiently as those with strong NA-binding affinity (such as PAMAM) after being immobilized onto the microparticle surface. This is important as it diminishes the dependence on highly charged polycations, which are often positively correlated with cytotoxicity. Immobilization of polycations also minimizes the systemic toxicity associated with their free forms, making the microparticles a safer therapeutic option. Furthermore, microparticles provide a large surface area, which allows complexation with larger ligands, such as cfNAs associated with vesicles or exosomes released upon cell death. They could function as a tissue ‘trash-processing station’ that allows enzymes such as DNase, RNase and protease to degrade the NA-containing complexes bound on the surface.

Inflammation is known to play a critical role in initiating tissue regeneration upon injury [52]. However, under chronic and other specific circumstances, inflammation could also lead to more tissue damage [53]. Increased secretion of metalloprotease not only facilitates the recruitment of immune cells but also leads to ECM degradation, cartilage and bone destruction, which is the primary underlying mechanism for osteoarthritis and rheumatoid arthritis [54, 55]. The high oxidative pressure in inflamed tissue can cause more cell death and tissue injury, which explains the damaging effect of inflammation in many ischemia/reperfusion injury [56]. In the models we studied, treatment with cationic microparticles significantly reduced the levels of inflammatory cytokines and neutrophil infiltration in the STS-induced peritonitis model. In the TMJ dysfunction model, microparticle treatment improved cartilage integrity and increased bone volume, indicating promotion of joint tissue repair. In the spinal cord injury model, microparticles downregulated oxidative stress markers such as iNOS, Cox2 and Nrf2, as well as cell death mediators including p53 and caspase 3. These results suggest that modulating tissue levels of DAMPs with cationic microparticles not only suppresses excessive inflammatory responses but also creates a microenvironment conducive to tissue regeneration after injury. Collectively, these statistically validated findings reinforce the conclusion that cationic microparticles effectively resolve inflammatory activity and promote structural recovery across multiple sterile injury models.

In addition to the therapeutic innovation, we also prepared cationic chitosan microparticles using microfluidics-prepared W/O/W double emulsions. For decades, innovation in materials synthesis for different applications has far preceded the advancement of material processing techniques [57]. For therapeutic development, with the same materials chemistry, fabrication may produce microparticles with different characteristics, such as size, shape, drug loading efficiency and release profile, which could all impact the therapeutic outcome. The fact that some of the polycations only functioned as cfNA scavengers in the immobilized form further illustrated that processing and fabrication could influence the biological outcome of biomaterials.

Microfluidics, which provides robust and precise manipulation of fluids, offers a powerful platform for microparticle processing and fabrication. Here, droplets containing chitosan solution were first prepared, and phase transition was induced to solidify the material. Osmotic pressure was then applied to condense the microparticles. As the microparticles were formed within the uniform double emulsion droplets, they displayed a narrow size distribution. Moreover, this fabrication method could facilitate the synthesis of composite chitosan microparticles that incorporate other biomaterials. It has the potential to precisely control the composition of the microparticles, as all the materials are contained in the same microenvironment in the double emulsion core. The control of microparticle synthesis is important because it could improve the colloidal stability and allow fine-tuning of the size and surface charge so that we can optimize the NA binding capacity, resistance to endocytosis, and *in vivo* biodistribution. Proper size control is essential, as micron-sized particles remain extracellular, reducing the risk of cellular uptake that could inadvertently trigger immune activation through TLR pathways. This design strategy prevents the reactivation of inflammation by avoiding the intracellular processing of adsorbed DAMPs.

The main strength of this work is that it proposes and validates a fundamentally different cfNA-scavenging strategy by immobilizing polycations on the surface of microparticles. This design not only enhances nucleic acid binding affinity but also prevents unintended cellular uptake. Another strength is the comprehensive evaluation across multiple sterile inflammation models, which provides robust evidence of therapeutic potential. Furthermore, the fabrication of chitosan-based microparticles using microfluidics represents a methodological advance, enabling precise control over particle size and uniformity, thereby improving translational relevance. However, this study also has limitations. First, the use of proof-of-concept models, while informative, may not fully recapitulate the complexity of chronic human inflammatory diseases. Second, polystyrene beads were used in the initial validation, but they are not biodegradable. Although chitosan microparticles were introduced as a biocompatible alternative, further *in vivo* testing with biodegradable systems is necessary before clinical translation. Meanwhile, whether microparticles have any direct effect on inflammation and TLR9 itself, as well as stability and binding kinetics of microparticles, should be evaluated in the future. Finally, the current work focused primarily on cfNA scavenging, and additional studies are needed to explore how this approach interacts with other DAMPs or immune pathways.

## Conclusion

Sterile inflammation driven by cfNA activation of innate immune receptors plays a key role in autoimmune diseases and tissue injuries. Targeting cfNA-induced inflammation has been challenging due to the complexity of TLR signaling and the lack of effective therapies. This study demonstrates that cfNAs from dying cells can be neutralized by polycation-coated microparticles. By coating microparticles with polycations, we enhanced their ability to bind cfNAs, preventing cellular uptake and reducing inflammatory responses. These microparticles reduced inflammation in models of acute peritonitis, TMJ dysfunction, and spinal cord injury by lowering cytokine levels, minimizing immune cell infiltration, and preserving tissue integrity. To improve robustness, we developed a microfluidics-based technique to produce uniform, chitosan-based microparticles with enhanced cfNA-scavenging properties. This method provided precise control over particle size, charge, and stability, optimizing therapeutic effects while minimizing toxicity. Our findings establish polycation-coated microparticles as a promising approach for regulating cfNA-driven inflammation and promoting tissue regeneration. Unlike conventional anti-inflammatory drugs and soluble or nanoparticulate scavengers, these microparticles act locally, reducing off-target risks and systemic toxicity.

## Materials and methods

Experimental details, including preparation of dying-cell supernatant, measurement of cfNAs, ninhydrin assay to quantify the amount of polymers coated on microparticle surface, scanning electron microscopy, ELISA assay for TNF-α and IL-6, RNA isolation and real-time PCR, and statistical analysis, are listed in the Supporting Information.

### TLR activation analysis

Ramos-Blue™ cells (InvivoGen, San Diego, CA, USA) were engineered to be responsive to NF-κB inducers, such as TLR3, TLR7, TLR9 and NOD1 agonists. When stimulated, they produce a soluble alkaline phosphatase (SEAP) in the supernatant that can be readily monitored by colorimetric methods in the presence of a substrate. Ramos-Blue™ cells were cultured in Iscove’s Modified Dulbecco’s Medium (IMDM) (Invitrogen) with 2 mM L-glutamine, 10% v/v heat-inactivated fetal bovine serum (FBS), Pen-Strep (50 U/mL and 50 μg/mL, respectively) and 100 μg/mL Zeocin™ (InvivoGen) as selection antibiotics.

For the TLR activation study, Ramos-Blue™ cells were resuspended in a fresh growth medium without Zeocin at a density of 2 × 10^6^ cells/mL. 180 μL cell suspension was seeded per well of a flat-bottom 96-well plate. 20 μL of TLR activators (concentration specified in the manuscript for different activators) were added after seeding. When evaluating the effect of microparticles or polymers, 10 μL of microparticle or polymer solution was first added to the cell suspension, which was immediately followed by the addition of 10 μL of the TLR activators. The plate was then incubated at 37°C with 5% CO_2_ for 24 h. To detect TLR activation, 160 μL QUANTI-BLUE™ (InvivoGen) was added per well of a flat-bottom 96-well plate, followed by 40 μL of supernatant from stimulated Ramos-Blue™ cells. The plate was then incubated for 4 h. SEAP activity was measured as absorbance at 650 nm using a plate reader (BMG Labtech, Ortenberg, Germany). TLR activators used for these studies were CpG 2006 (InvivoGen), poly(I:C) (InvivoGen), CpG 2006 with F7-26 (Enzo Life Sciences, Farmingdale, NY, USA), STS-treated dying-cell supernatant, and lupus patient plasma (Plasma service group, Southampton, PA, USA).

### Cationic polystyrene microparticle preparation

Polystyrene beads bearing surface carboxyl groups were coated with polycations by incubating them in a 2 mg/mL polycation solution for 30 min at room temperature. Following incubation, the beads were washed three times with phosphate-buffered saline (PBS) through repeated centrifugation and resuspension to remove unbound polycation. Successful coating was confirmed by measuring the Zeta potential, which showed a shift consistent with surface charge reversal, indicating effective polycation adsorption.

### Microfluidics-mediated chitosan microparticle formation

Fabrication and treatment of PDMS chips for double emulsion generation were described earlier [42]. Chip 1 and Chip 2 were made to smaller dimensions to form smaller droplets. 1% w/v Protasan™ chitosan (MW∼380kDa, 90% deacetylation) (NovaMatrix, Sandvika, Norway) dissolved in 100 mM sodium acetate buffer (pH 5.2) was used as the inner phase for double emulsion formation. Perfluorocarbon Novec™ HFE7500 engineered fluid (3 M, St. Paul, MN, USA) was used as the oil phase and served as a barrier to prevent out-diffusion of chitosan. 1% Picosurf 2 (Dolomite Microfluidics, Royston, UK) was used as the surfactant for the oil phase. The continuous phase was made of 1% Pluronic F127 (Sigma Aldrich) dissolved in the same buffer as chitosan. To control the final size of the microparticles, the relative flow rates of the three phases were adjusted to form double emulsions with inner cores of different sizes. Double emulsion droplets were collected into a 15 mL Falcon tube and transferred into a 10 wt% Glycine (pH 10) solution. The volume of the basic solution was 10 times that of the droplet solution. The tube was left on an orbiter shaker (Labnet International, Edison, NJ, USA) under gentle shaking at 20 rpm overnight. The upper glycine solution was carefully removed without disturbing the bottom droplet layer. After that, an equal volume of 1H, 1H, 2H, 2H-perfluoro-1-octanol (Sigma Aldrich) was added to rupture the droplets. Released particles were washed with PBS three times by repeated centrifugation and resuspension. The particles were then immersed in 2 mg/mL heparin (Merck Millipore, Billerica, MA, USA) solution for 30 min. After washing three times with PBS, the microparticles were resuspended in solutions containing 2 mg/mL cationic polymers for 30 min and washed. The coated microparticles were stable after resuspending in water or PBS at room temperature. Polystyrene beads with surface carboxyl groups (Polysciences, Warrington, PA, USA) were coated similarly in the same cationic polymer solution. Zeta potential was measured with a Zetasizer NanoZS-90 (Malvern Instruments, Southborough, MA, USA).

For fluorescence microscope imaging of the microparticles, FITC-labeled dextran (10 kDa, Sigma Aldrich) was incorporated into the chitosan core. Cy5-labeled PAMAM was used to coat the outer layer. Fluorescence images of microparticles were captured with an inverted fluorescence microscope (TE2000U, Nikon Instruments, Melville, NY, USA) equipped with a 100-W mercury lamp (X-Cite 120 Fluor system, EXFO, ON, Canada) and a cooled CCD (CoolSnap HQ, Roper Scientific, Tucson, AZ, USA).

### Flow cytometry

Flow cytometric analysis was conducted via a FACSCanto II (BD Biosciences, Franklin Lakes, NJ, USA). A minimum of 10 000 cells were analyzed per sample. For the cellular uptake study, DC2.4 cells cultured in DMEM (Invitrogen) with 10% FBS and Pen-Strep (50 U/mL and 50 μg/mL, respectively) were seeded at 2 × 10^5^ cells/mL per well of a 24-well plate. Cells were washed with PBS 20 h after seeding, and 450 μL OptiMEM was added to each well. 25 μL of 10 μg/mL PS, microparticles (1 × 10^6^ microparticles/mL), or PBS was added to the cells. 25 μL of 100 μM AlexaFluor-488-labeled CpG (5 μM final concentration) (IDT, Coralville, IA) or PBS as a negative control was then added to the cells. Cells were washed 4 h or 24 h after CpG addition, trypsinized, and fixed with 4% Paraformaldehyde (Sigma Aldrich) in PBS for 15 min and stored at 4°C for flow cytometry analysis. Data were analyzed, and graphs were plotted using FlowJo (Ashland, OR, USA).

For the *in vivo* study, after pelleting cells from the peritoneal lavage, the cells were immediately suspended in a hypotonic lysis buffer (Biolegend, San Diego, CA, USA) for 5 min to remove red blood cells. Followed by centrifugation and washing with PBS containing 3% FBS, cells were counted, and cell density was adjusted to 1 × 10^7^/mL in PBS with 3% FBS. 200 µl of cells were aliquoted for antibody staining. The cells were first incubated with anti-mouse CD16/CD32 antibody (Biolegend) for 20 min to block Fc receptors. FITC-anti-mouse Ly-6G (Biolegend) was then added to stain neutrophils. The cells were then washed with PBS and fixed with 4% paraformaldehyde and stored at 4°C for flow cytometry analysis. Neutrophil number was determined by multiplying the total cell numbers by the percentage of Ly6G-positive cells.

### STS-induced acute peritonitis *in vivo*

C57BL/6 mice (male, 8 weeks old) were used to induce acute peritonitis, and all procedures complied with Duke University’s Institutional Animal Care and Use Committee. C57BL/6 mice were intraperitoneally injected with 0.3 mg/kg of STS (or as otherwise indicated) in 0.2 mL of 0.9% NaCl using an ultra-fine insulin syringe (BD, Franklin Lakes, NJ, USA). Equal volumes of 0.9% NaCl were injected as negative controls. Free PS (20 mg/kg) (STS + Polymer group), or PBS solution (PBS group) was intraperitoneally injected 2 h after STS injection. 0.2 mL PS-coated microparticles (5 × 10^7^/mL) were used as single- or multi-dose, including 2 h after STS injection (STS + Microparticle 1), and 2 and 6 h after STS injection (STS + Microparticle 2). Animals were euthanized by CO_2_ exposure 16 h post STS injection. 5 mL cold and sterile PBS was injected into the peritoneal cavity with a 27 G syringe (BD, Franklin Lakes, NJ, USA), and the mice were massaged to dislocate cells in the peritoneal space. The peritoneal lavage was then carefully withdrawn from the mice and collected into a 15 mL tube. The peritoneal lavages were centrifuged at 500 g for 5 min, and supernatants were stored at −20°C until analyzed for cytokines. Cell pellets were taken to prepare for immune staining and flow cytometry study (see flow cytometry analysis).

### Temporomandibular joint dysfunction model

Sprague-Dawley rats (male, 8 weeks old) were used to establish a temporomandibular joint (TMJ) osteoarthritis model, and all procedures complied with the Dankook University’s Institutional Animal Care and Use Committee (Approval No. DKU-15). Rats were anesthetized by intramuscular injection of ketamine (80 mg/kg) and xylazine (10 mg/kg), followed by intraarticular injection of 20 µL complete Freund’s adjuvant (CFA, 15 µg/mL) into the superior joint space using a 30-gauge Hamilton syringe. Three rats were initially examined to confirm the successful induction of the defect model.

A healthy (non-defect) group and a CFA-induced defect group treated with saline were used as positive and negative controls, respectively. Microparticles of two different compositions (polycation-coated polystyrene and chitosan) were injected into the TMJ space at three different doses (0.5 × 10^6^, 1 × 10^6^ and 5 × 10^6^ microparticles/mL). The PS solution, used as the dispersant for microparticles, was also injected as one experimental control. Three days after the CFA injection, each test group was injected at 20 µL into the TMJ space. Each group consisted of 4–5 rats, and a total of 35 rats were used for the experiments.

Ten days after the injection, animals were sacrificed, and the samples were collected in a neutralized buffered saline solution. After 24 h of fixation, samples were scanned using a micro-CT scanner (Skyscan 1176, Skyscan, Belgium) at a camera pixel size of 12.56 µm using an X-ray with a current of 385 µA, a voltage of 65 kV, and an exposure time of 279 ms. The 3D and 2D projection images were reconstructed through a computer analysis program (CTAn Skyscan), and the bone volume (mm^3^) of the TMJ was calculated by choosing a cylindrical region of interest (ROI).

For the histological analysis, the samples were decalcified with RapidCal™ solution (BC Chemical CO, Stanwood, WA, USA) and dehydrated with a series of graded ethanol solutions (70%, 90%, 95% and 100%). Samples were embedded in paraffin and then cut to approximately 5 µm thick sections using a microtome (RM2245, Leica, Germany). Samples were then stained with Hematoxylin and Eosin (H&E) by a routine technique and then examined under an optical microscope (IX71, Olympus, Japan).

For the DNA staining, the samples were prepared in blocks by embedding in paraffin, which were cut into 5 mm-thick sections using a microtome (LEICATM). Tissue sections were deparaffinized at 60°C for 2 h and hydrated through graded ethanol solutions, which were then stained with NucRed^®^ Dead 647 ReadyProbes^®^ Reagent (Life Technologies), following the manufacturer’s instructions. The Reagent-stained DNAs of/from dead cells, and emitted bright far-red fluorescence when bound to DNA. Stained samples were evaluated under fluorescent microscopy (IX71, Olympus, Japan).

### *In vivo *models of spinal cord contusion and local delivery of microparticles

Adult female Sprague-Dawley rats (12-week-old, 230–250 g) were used, and all procedures complied with Dankook University’s Institutional Animal Care and Use Committee (Approval No. DKU-15). Surgical procedures have been previously described in detail [58]. Briefly, rats were deeply anesthetized by isoflurane (Forane; Choongwae Pharma, Seoul, Korea) inhalation, and a laminectomy was performed at the T9-T10 level. All animals received a moderate contusion injury (200kdyne) to expose the T9 spinal cord using the Infinite Horizon impactor (IH-400, Precision Systems and Instrumentation, LLC, KY, USA). PS-coated microparticles with different concentrations (0.5 × 10^6^, 1 × 10^6^ and 5 × 10^6^  microparticles/10 μL) were prepared immediately before use and were suspended in distilled water. At 30 min following contusion, a total volume of 10 μL PS-coated microparticles solution during 10 min was directly delivered to the lesion site via Hamilton syringe at a rate of 1 µl/min (Hamilton Company, Reno, NV, USA). Control animals received the same amount of PBS. After delivering the solution, the cord was then covered with a piece of hemostatic agent (Surgicel^®^, Johnson and Johnson, Arlington, TX, USA), and the muscle and subcutaneous layers, skin were closed. Intramuscular injection of 40 mg/kg cefotiam hydrochloride (Fontiam™, Hanmi Pharma, Seoul, Korea) was performed in all operated rats for 3 days, and intraperitoneal injection of normal saline (3 mL) was done just after surgery. Animals also received oral administration of 10 mg/kg acetaminophen syrup (Tylenol™, Janssen Pharmaceutica, Titusville, NJ, USA) for 3 days, and bladder expression was performed two times per day and continued until the amount of expressed urine was less than 0.5 mL/day. These groups of rats were sacrificed at 1 week following spinal cord injury (*n* = 7 per group).

Frozen sections were used for H&E staining and immunohistochemistry (IHC) analysis. Rats (*n* = 4 per group) were perfused with 0.9% saline followed by 4% paraformaldehyde (Hushi Inc., Shanghai, China) in 0.1 M PBS (pH 7.4). The spinal cord was then dissected, post-fixed overnight in 4% paraformaldehyde at 4°C, and transferred to 30% sucrose in 0.1 M PBS for 3 days. The cord was embedded in M1 compound (Thermo Fisher Scientific Inc.) and cryosectioned at 16 µm in the sagittal plane.

H&E staining was performed to detect the lesion site of the injured spinal cord at 1 week post-injury. The sections were stained with hematoxylin for 5 min, rinsed in running tap water for 3 min, and then stained with eosin for 1 min. The stained sections were dehydrated through graded ethanol, cleared with xylene, and then imaged under a light microscope (Nikon, Tokyo, Japan).

IHC was used to analyze the inflammatory response in the contused spinal cord. The primary antibodies, rabbit anti-GFAP (1:1000, Dako) and mouse anti-monocyte/macrophage CD68 (1:400, Millipore), were incubated overnight at 4°C. After the sections were washed three times, goat anti-rabbit (Alexa Fluor 546, Jackson ImmunoResearch) and goat anti-mouse (Alexa Fluor 488, Jackson ImmunoResearch) secondary antibodies were used at a dilution of 1:200 in 2% NGS in PBS. Following 2 h incubation, the sections were washed three times with PBS. Stained tissue sections were imaged using a confocal microscope (Carl Zeiss Inc.). For quantification of the lesion cavity size in the sagittal section, three representative images from the lesion site per animal (*n* = 4 per group) were captured and analyzed using Image J software (1.37 v, National Institutes of Health). For quantitation of CD68^+^ monocytes and macrophages in the sagittal section, the images were captured at the lesion site (*n* = 4 per group) using 100× magnification on a confocal microscope for manual cell counts and expressed cell numbers per 1 mm^2^.

## Supplementary Material

rbaf135_Supplementary_Data

## References

[1] Chen GY , Nuñez G. Sterile inflammation: sensing and reacting to damage. Nat Rev Immunol 2010;10:826–37. 21088683 10.1038/nri2873 PMC3114424

[2] Ma M , Jiang W, Zhou R. DAMPs and DAMP-sensing receptors in inflammation and diseases. Immunity 2024;57:752–71. 38599169 10.1016/j.immuni.2024.03.002

[3] Wang K , Huang H, Zhan Q, Ding H, Li Y. Toll-like receptors in health and disease. MedComm (2020) 2024;5:e549. (2020) 38685971 10.1002/mco2.549 PMC11057423

[4] Pös O , Biró O, Szemes T, Nagy B. Circulating cell-free nucleic acids: characteristics and applications. Eur J Hum Genet 2018;26:937–45. 29681621 10.1038/s41431-018-0132-4 PMC6018748

[5] Cheng X , Sui H, Chen F, Li C, Du M, Zhang S, Chen J, Dou J, Huang Y, Xie X, Cheng C, Yang R, Yang C, Shi B, Shao D, Leong KW, Huang H. Nanomaterial-Mediated reprogramming of macrophages to inhibit refractory muscle fibrosis. Adv Mater 2024;36:2410368. 10.1002/adma.202410368 PMC11849413 39548911

[6] Relja B , Land WG. Damage-associated molecular patterns in trauma. Eur J Trauma Emerg Surg 2020;46:751–75. 31612270 10.1007/s00068-019-01235-w PMC7427761

[7] Land WG , Agostinis P, Gasser S, Garg AD, Linkermann A. Transplantation and damage-associated molecular patterns (DAMPs). Am J Transplant 2016;16:3338–61. 27421829 10.1111/ajt.13963

[8] Mukherjee S , Bayry J. The Yin and Yang of TLR4 in COVID-19. Cytokine Growth Factor Rev 2025;82:70–85. 39490235 10.1016/j.cytogfr.2024.10.001

[9] Behzadi P , Chandran D, Chakraborty C, Bhattacharya M, Saikumar G, Dhama K, Chakraborty A, Mukherjee S, Sarshar M. The dual role of toll-like receptors in COVID-19: balancing protective immunity and immunopathogenesis. Int J Biol Macromol 2025;284:137836. 39613064 10.1016/j.ijbiomac.2024.137836

[10] Mukherjee S , Patra R, Behzadi P, Masotti A, Paolini A, Sarshar M. Toll-like receptor-guided therapeutic intervention of human cancers: molecular and immunological perspectives. Front Immunol 2023;14:1244345. 37822929 10.3389/fimmu.2023.1244345 PMC10562563

[11] Behzadi P , Sameer AS, Nissar S, Banday MZ, Gajdács M, García-Perdomo HA, Akhtar K, Pinheiro M, Magnusson P, Sarshar M, Ambrosi C. The interleukin-1 (IL-1) superfamily cytokines and their single nucleotide polymorphisms (SNPs). J Immunol Res 2022;2022:2054431. 35378905 10.1155/2022/2054431 PMC8976653

[12] Hei Y , Du J, Deng Z, Deng Y, Guan Y, Yang J, Chen S, Zhang Z, Jiang S, Zhang Q. Therapeutic effects of PEG-modified polyamide amine dendrimer for cell free DNA adsorption in temporomandibular joint osteoarthritis. ACS Appl Mater Interfaces 2024;16:39153–64. 39018481 10.1021/acsami.4c08569

[13] Shi T , Zhao J, Long K, Gao M, Chen F, Chen X, Zhang Y, Huang B, Shao D, Yang C, Wang L, Zhang M, Leong KW, Chen L, He K. Cationic mesoporous silica nanoparticles alleviate osteoarthritis by targeting multiple inflammatory mediators. Biomaterials 2023;303:122366. 37948854 10.1016/j.biomaterials.2023.122366

[14] Zhu H , Huang D, Nie M, Zhao Y, Sun L. Dexamethasone loaded DNA scavenger nanogel for systemic lupus erythematosus treatment. Bioact Mater 2025;43:330–9. 40115883 10.1016/j.bioactmat.2024.08.030 PMC11923376

[15] Tu Z , Zhong Y, Hu H, Shao D, Haag R, Schirner M, Lee J, Sullenger B, Leong KW. Design of therapeutic biomaterials to control inflammation. Nat Rev Mater 2022;7:557–74. 35251702 10.1038/s41578-022-00426-z PMC8884103

[16] Liang H , Peng B, Dong C, Liu L, Mao J, Wei S, Wang X, Xu H, Shen J, Mao HQ, Gao X, Leong KW, Chen Y. Cationic nanoparticle as an inhibitor of cell-free DNA-induced inflammation. Nat Commun 2018;9:4291. 30327464 10.1038/s41467-018-06603-5 PMC6191420

[17] Dawulieti J , Sun M, Zhao Y, Shao D, Yan H, Lao YH, Hu H, Cui L, Lv X, Liu F, Chi CW, Zhang Y, Li M, Zhang M, Tian H, Chen X, Leong KW, Chen L. Treatment of severe sepsis with nanoparticulate cell-free DNA scavengers. Sci Adv 2020;6:eaay7148. 32523983 10.1126/sciadv.aay7148 PMC7259927

[18] Shi C , Dawulieti J, Shi F, Yang C, Qin Q, Shi T, Wang L, Hu H, Sun M, Ren L, Chen F, Zhao Y, Liu F, Li M, Mu L, Liu D, Shao D, Leong KW, She J. A nanoparticulate dual scavenger for targeted therapy of inflammatory bowel disease. Sci Adv 2022;8:eabj2372. 35089791 10.1126/sciadv.abj2372 PMC8797786

[19] Zhu J , Li X, Zhou Y, Ge C, Li X, Hou M, Wei Y, Chen Y, Leong KW, Yin L. Inhaled immunoantimicrobials for the treatment of chronic obstructive pulmonary disease. Sci Adv 2024;10:eabd7904. 38324682 10.1126/sciadv.abd7904 PMC10849584

[20] Huang H , Pan W, Wang Y, Kim HS, Shao D, Huang B, Ho TC, Lao YH, Quek CH, Shi J, Chen Q, Shi B, Zhang S, Zhao L, Leong KW. Nanoparticulate cell-free DNA scavenger for treating inflammatory bone loss in periodontitis. Nat Commun 2022;13:5925. 36207325 10.1038/s41467-022-33492-6 PMC9546917

[21] Li T , Akinade T, Zhou J, Wang H, Tong Q, He S, Rinebold E, Valencia Salazar LE, Bhansali D, Zhong Y, Ruan J, Du J, Dalerba P, Leong KW. Therapeutic nanocarriers inhibit chemotherapy-induced breast cancer metastasis. Adv Sci (Weinh) 2022;9:e2203949. 36220339 10.1002/advs.202203949 PMC9685442

[22] Shen H , Xu B, Yang C, Xue W, You Z, Wu X, Ma D, Shao D, Leong K, Dai J. A DAMP-scavenging, IL-10-releasing hydrogel promotes neural regeneration and motor function recovery after spinal cord injury. Biomaterials 2022;280:121279. 34847433 10.1016/j.biomaterials.2021.121279

[23] Chen X , Huang H, Guo C, Zhu X, Chen J, Liang J, Yang R, Shao D, Chen F, Shi B, Yang C, Leong KW, Zhao L. Controlling alveolar bone loss by hydrogel-based mitigation of oral dysbiosis and Bacteria-triggered proinflammatory immune response. Adv Funct Materials 2025;35:2409121.

[24] Yang C , Dawulieti J, Zhang K, Cheng C, Zhao Y, Hu H, Li M, Zhang M, Chen L, Leong KW, Shao D. An injectable antibiotic hydrogel that scavenges proinflammatory factors for the treatment of severe abdominal trauma. Adv Funct Materials 2022;32:2111698.

[25] Kaczanowska S , Joseph AM, Davila E. TLR agonists: our best frenemy in cancer immunotherapy. J Leukoc Biol 2013;93:847–63. 23475577 10.1189/jlb.1012501 PMC3656332

[26] Krieg AM. Toll-like receptor 9 (TLR9) agonists in the treatment of cancer. Oncogene 2008;27:161–7. 18176597 10.1038/sj.onc.1210911

[27] Gill R , Ruan X, Menzel CL, Namkoong S, Loughran P, Hackam DJ, Billiar TR. Systemic inflammation and liver injury following hemorrhagic shock and peripheral tissue trauma involve functional TLR9 signaling on bone marrow-derived cells and parenchymal cells. Shock 2011;35:164–70. 20577143 10.1097/SHK.0b013e3181eddcab PMC3000874

[28] Staak C , Salchow F, Clausen PH, Luge E. Polystyrene as an affinity chromatography matrix for the purification of antibodies. J Immunol Methods 1996;194:141–6. 8765167 10.1016/0022-1759(96)00142-1

[29] Lee J , Sohn JW, Zhang Y, Leong KW, Pisetsky D, Sullenger BA. Nucleic acid-binding polymers as anti-inflammatory agents. Proc Natl Acad Sci U S A 2011;108:14055–60. 21844380 10.1073/pnas.1105777108 PMC3161575

[30] Jain S , Pitoc GA, Holl EK, Zhang Y, Borst L, Leong KW, Lee J, Sullenger BA. Nucleic acid scavengers inhibit thrombosis without increasing bleeding. Proc Natl Acad Sci U S A 2012;109:12938–43. 22837404 10.1073/pnas.1204928109 PMC3420207

[31] Means TK , Latz E, Hayashi F, Murali MR, Golenbock DT, Luster AD. Human lupus autoantibody-DNA complexes activate DCs through cooperation of CD32 and TLR9. J Clin Invest 2005;115:407–17. 15668740 10.1172/JCI23025 PMC544604

[32] Williamson RA , Burgoon MP, Owens GP, Ghausi O, Leclerc E, Firme L, Carlson S, Corboy J, Parren PW, Sanna PP, Gilden DH, Burton DR. Anti-DNA antibodies are a major component of the intrathecal B cell response in multiple sclerosis. Proc Natl Acad Sci U S A 2001;98:1793–8. 11172030 10.1073/pnas.031567598 PMC29336

[33] Choi JJ , Reich CF 3rd, Pisetsky DS. Release of DNA from dead and dying lymphocyte and monocyte cell lines in vitro. Scand J Immunol 2004;60:159–66. 15238085 10.1111/j.0300-9475.2004.01470.x

[34] Jahr S , Hentze H, Englisch S, Hardt D, Fackelmayer FO, Hesch RD, Knippers R. DNA fragments in the blood plasma of cancer patients: quantitations and evidence for their origin from apoptotic and necrotic cells. Cancer Res 2001;61:1659–65. 11245480

[35] Krysko DV , Kaczmarek A, Krysko O, Heyndrickx L, Woznicki J, Bogaert P, Cauwels A, Takahashi N, Magez S, Bachert C, Vandenabeele P. TLR-2 and TLR-9 are sensors of apoptosis in a mouse model of doxorubicin-induced acute inflammation. Cell Death Differ 2011;18:1316–25. 21311566 10.1038/cdd.2011.4 PMC3172099

[36] Guo Z , Tang N, Liu FY, Yang Z, Ma SQ, An P, Wu HM, Fan D, Tang QZ. TLR9 deficiency alleviates doxorubicin-induced cardiotoxicity via the regulation of autophagy. J Cell Mol Med 2020;24:10913–23. 33140921 10.1111/jcmm.15719 PMC7521247

[37] Xiang T , Tao ZY, Liao LF, Wang S, Cao DY. Animal models of temporomandibular disorder. J Pain Res 2021;14:1415–30. 34079358 10.2147/JPR.S303536 PMC8166243

[38] Hellenbrand DJ , Quinn CM, Piper ZJ, Morehouse CN, Fixel JA, Hanna AS. Inflammation after spinal cord injury: a review of the critical timeline of signaling cues and cellular infiltration. J Neuroinflammation 2021;18:284. 34876174 10.1186/s12974-021-02337-2 PMC8653609

[39] Kigerl KA , Popovich PG. Toll-like receptors in spinal cord injury. Curr Top Microbiol Immunol 2009;336:121–36. 19688331 10.1007/978-3-642-00549-7_7

[40] Agnihotri SA , Mallikarjuna NN, Aminabhavi TM. Recent advances on chitosan-based micro- and nanoparticles in drug delivery. J Control Release 2004;100:5–28. 15491807 10.1016/j.jconrel.2004.08.010

[41] He P , Davis SS, Illum L. Chitosan microspheres prepared by spray drying. Int J Pharm 1999;187:53–65. 10502613 10.1016/s0378-5173(99)00125-8

[42] Zhang Y , Ho YP, Chiu YL, Chan HF, Chlebina B, Schuhmann T, You L, Leong KW. A programmable microenvironment for cellular studies via microfluidics-generated double emulsions. Biomaterials 2013;34:4564–72. 23522800 10.1016/j.biomaterials.2013.03.002 PMC3649128

[43] Yeager MP , Guyre PM, Munck AU. Glucocorticoid regulation of the inflammatory response to injury. Acta Anaesthesiol Scand 2004;48:799–813. 15242423 10.1111/j.1399-6576.2004.00434.x

[44] Guiducci C , Gong M, Xu ZH, Gill M, Chaussabel D, Meeker T, Chan JH, Wright T, Punaro M, Bolland S, Soumelis V, Banchereau J, Coffman RL, Pascual V, Barrat FJ. TLR recognition of self nucleic acids hampers glucocorticoid activity in lupus. Nature 2010;465:937–41. 20559388 10.1038/nature09102 PMC2964153

[45] Wang D , Bhagat L, Yu D, Zhu FG, Tang JX, Kandimalla ER, Agrawal S. Oligodeoxyribonucleotide-based antagonists for toll-like receptors 7 and 9. J Med Chem 2009;52:551–8. 19102653 10.1021/jm8014316

[46] Capolunghi F , Rosado MM, Cascioli S, Girolami E, Bordasco S, Vivarelli M, Ruggiero B, Cortis E, Insalaco A, Fanto N, Gallo G, Nucera E, Loiarro M, Sette C, De Santis R, Carsetti R, Ruggiero V. Pharmacological inhibition of TLR9 activation blocks autoantibody production in human B cells from SLE patients. Rheumatology (Oxford) 2010;49:2281–9. 20739362 10.1093/rheumatology/keq226

[47] Jackman JG , Juwarker H, Poveromo LP, Levinson H, Leong KW, Sullenger BA. Polycationic nanofibers for nucleic acid scavenging. Biomacromolecules 2016;17:3706–13. 27741396 10.1021/acs.biomac.6b01236

[48] Lee J , Jackman JG, Kwun J, Manook M, Moreno A, Elster EA, Kirk AD, Leong KW, Sullenger BA. Nucleic acid scavenging microfiber mesh inhibits trauma-induced inflammation and thrombosis. Biomaterials 2017;120:94–102. 28049065 10.1016/j.biomaterials.2016.12.024 PMC5260826

[49] Wan Q , Huang B, Li T, Xiao Y, He Y, Du W, Wang BZ, Dakin GF, Rosenbaum M, Goncalves MD, Chen S, Leong KW, Qiang L. Selective targeting of visceral adiposity by polycation nanomedicine. Nat Nanotechnol 2022;17:1311–21. 36456644 10.1038/s41565-022-01249-3

[50] Huang B , Wan Q, Li T, Yu L, Du W, Calhoun C, Leong KW, Qiang L. Polycationic PAMAM ameliorates obesity-associated chronic inflammation and focal adiposity. Biomaterials 2023;293:121850. 36450630 10.1016/j.biomaterials.2022.121850 PMC12433583

[51] Porello I , Bono N, Candiani G, Cellesi F. Advancing nucleic acid delivery through cationic polymer design: non-cationic building blocks from the toolbox. Polym Chem 2024;15:2800–26.

[52] Cooke JP. Inflammation and its role in regeneration and repair. Circ Res 2019;124:1166–8. 30973815 10.1161/CIRCRESAHA.118.314669 PMC6578588

[53] Chen L , Deng H, Cui H, Fang J, Zuo Z, Deng J, Li Y, Wang X, Zhao L. Inflammatory responses and inflammation-associated diseases in organs. Oncotarget 2018;9:7204–18. 29467962 10.18632/oncotarget.23208 PMC5805548

[54] Orlowsky EW , Kraus VB. The role of innate immunity in osteoarthritis: when our first line of defense goes on the offensive. J Rheumatol 2015;42:363–71. 25593231 10.3899/jrheum.140382 PMC4465583

[55] Goh FG , Midwood KS. Intrinsic danger: activation of toll-like receptors in rheumatoid arthritis. Rheumatology (Oxford) 2012;51:7–23. 21984766 10.1093/rheumatology/ker257

[56] Gill R , Tsung A, Billiar T. Linking oxidative stress to inflammation: toll-like receptors. Free Radic Biol Med 2010;48:1121–32. 20083193 10.1016/j.freeradbiomed.2010.01.006 PMC3423196

[57] Zhang Y , Chan HF, Leong KW. Advanced materials and processing for drug delivery: the past and the future. Adv Drug Deliv Rev 2013;65:104–20. 23088863 10.1016/j.addr.2012.10.003 PMC3565095

[58] Park WB , Kim SY, Lee SH, Kim HW, Park JS, Hyun JK. The effect of mesenchymal stem cell transplantation on the recovery of bladder and hindlimb function after spinal cord contusion in rats. BMC Neurosci 2010;11:119. 20846445 10.1186/1471-2202-11-119 PMC2955046

