# Supplementary material for: Cationic microparticles inhibit local sterile inflammation in tissue injuries
Source: Regen Biomater. 2025 Dec 31;13:rbaf135. doi: 10.1093/rb/rbaf135 (PMC12900541; doi:10.1093/rb/rbaf135)
Supplement: rbaf135_Supplementary_Data [file rbaf135_supplementary_data.zip › Revision_SI_Cationic Microparticles Inhibit Sterile Inflammation in Tissue Injuries_clean_26 Dec 2026.docx]

**Supplemental Information**

Cationic Microparticles Inhibit Sterile Inflammation in Tissue Injuries

**Supplemental Materials and Methods**

***Preparation of dying cell supernatant***

Cells were seeded at 5×10^5^ cells/ml and cultured at 37°C and 5% CO_2_ in the appropriate complete growth media according to the supplier. When the cell density reached about 80% confluence, cells were washed with PBS and cultured in serum- and antibiotic-free Opti-MEM (Invitrogen, Carlsbad, CA). Staurosporine (STS) and Doxorubicin (DOX) (Sigma Aldrich, St Louis, MO) dissolved in PBS were added at specified concentrations. After 2 hours, treated cells were washed 5 times with PBS to remove excessive drugs in the culture medium. Fresh Opti-MEM was then added to the treated cells. 24 hours after drug treatment, cell suspension was collected and centrifuged at 500 g for 5 minutes to remove large cell debris. The supernatant was directly used for subsequent analysis.

For the nuclease degradation of NAs, DNase I (40 U/ml) (Invitrogen), RNase A (10 μg/ml) (Invitrogen), or both were added to the dying cell supernatant and incubated at 37°C for 30 minutes. The supernatant was then heated at 75°C for 5 minutes to inactive the enzymes. To inactivate the proteins in the dying cell supernatant, the solution was heated at 65°C for 30 minutes. The supernatant was then applied to reporter cells to analyze the pro-inflammatory properties.

***Measurement of cfNAs***

DNA/RNA concentration was assessed by a fluorimetric assay as described previously [37]. Briefly, sample in various dilutions were mixed at a 1:1 ratio with the dye PicoGreen/RiboGreen (Molecular Probes, Eugene, OR), diluted 1:200 in 10 mM Tris, 1 mM EDTA, pH 8 (TE buffer) in a black, 96-well microtiter plate (Costar, Corning, NY). The DNA concentration was determined from fluorescence measurements using a BMG Labtech FLUOStar Optima plate reader (BMG Labtech, Ortenberg, Germany) at 485 nm/535 nm excitation/emission wavelength. Data were collected as relative fluorescence units. The concentration of DNA in supernatant or peritoneum fluid was calculated according to a standard curve using double-stranded calf thymus DNA (Sigma Aldrich). Peritoneal fluids were diluted 1:10 in TE buffer, and DNA levels were measured as described above.

To evaluate the binding of microparticles with NAs, microparticles at different concentrations were added to 6 μM CpG solution and allowed to complex for 15 minutes. The solution was then centrifuged at 2000 g for 5 minutes. Free CpG concentration in the supernatant was determined using the same procedure described above with a standard curve using CpG DNA.

***Ninhydrin assay to quantify amount of polymers coated on microparticle surface***

The amount of polymers coated on the microparticle surface was determined by measuring polymer concentration before and after being coated onto the microparticles. The polymer concentration is determined by measuring free amino groups in the solution. 50 μl 3 mg/ml Fluorescamine (Sigma Aldrich) and 150 μl polymer solution (before and after being coated) was added at 1:3 volume ratio into a 96-well plate and incubated at room temperature for 15 minutes. Relative fluorescence intensity was measured using a plate reader at 360 nm/470 nm excitation/emission wavelength. The polymer concentration was determined by intrapolating into a standard curve of made for the same polycation.

***Scanning electron microscopy (SEM)***

The microparticles were sputter-coated with a gold layer of 10 nm thick using a Denton Vacuum Desk IV sputter unit at 75 mTor and 18 mA (Denton Vacuum, LLC, Moorestown, NJ, USA). SEM micrographs were obtained from a FEI XL30 SEM-FEG (FEI Co., Hillsboro, OR, USA). SEM images were imported into ImageJ to analyze particle size. Aggregates were excluded or manually outlined to ensure only individual particles were measured. Measurements were exported to plot the size distribution.

***ELISA assay for TNF-α and IL-6***

Cytokine concentration in supernatant or peritoneal lavage was determined using ELISA kit for mouse TNF-α and IL-6 (Life technologies) following suppliers’ protocol.

***RNA isolation and real-time PCR***

Total RNA (n=3 per group) was extracted from spinal cord by using an RNeasy mini kit (Qiagen, Hilden, Germany). cDNA was synthesized using random hexamer primers and SuperScript III (Invitrogen, Thermo Fisher Scientific Inc.). All primers pairs were designed using the UCSC Genome Bioinformatics and the NCBI database and are listed in Table S1. Real-time PCR was performed using Fast SYBR Green Master Mix (Applied Biosystems, Thermo Fisher Scientific Inc.) on a StepOne Real-Time PCR system (Thermo Fisher Scientific Inc.). Each real-time PCR was performed on at least triplicate assay. And expression of each target gene was normalized to GAPDH and expressed as the fold change relative to the control groups.

Statistical Analysis

All numeric data are reported as means ± SEM, and IBM SPSS Statistics 21 (International Business Machines Corp., Armonk, NY, USA) was used for analysis. The Shapiro-Wilk test was performed to check normal distribution of all quantified data from each group, and according to the result, Mann-Whitney *U* test was used to detect the differences between control and various concentration of experimental groups.

**Table S1. Primer sequences used for real time PCR**

| **Gene** | **5’-3’** | **Primer sequence** |
| --- | --- | --- |
| iNOS | Forward | CTCAGCACAGAGGGCTCAAAG |
|  | Reverse | TGCACCCAAACACCAAGGT |
| COX-2 | Forward | GGCCATGGAGTGGACTTAAA |
|  | Reverse | CTCTCCACCGATGACCTGAT |
| Nrf2 | Forward | TCCAGACAGACACCAGTGGA |
|  | Reverse | GGAATGTCTCTGCCAAAAGC |
| p53 | Forward | ACAGCGTGGTGGTACCGTAT |
|  | Reverse | GGAGCTGTTGCACATGTACT |
| Casp 3 | Forward | GAACGCGAAGAAAAGTGACC |
|  | Reverse | GAGTCCATCGACTTGCTTCC |
| IL-1β | Forward | GCCCGTCCTCTGTGACTCGT |
|  | Reverse | TGTCGTTGCTTGTCTCTCCTTGTA |
| IL-6 | Forward | ACCACCCACAACAGACCAGT |
|  | Reverse | CAGAATTGCCATTGCACAAC |
| IL-10 | Forward | CAGCTGCGACGCTGTCATCG |
|  | Reverse | GCAGTCCAGTAGATGCCGGGT |
| TNF-α | Forward | CTCAAGCCCTGGTATGAGCC |
|  | Reverse | GGCTGGGTAGAGAACGGATG |
| GAPDH | Forward | CACTGAGCATCTCCCTCACA |
|  | Reverse | GAGGGTGCAGCGAACTTTAT |

**
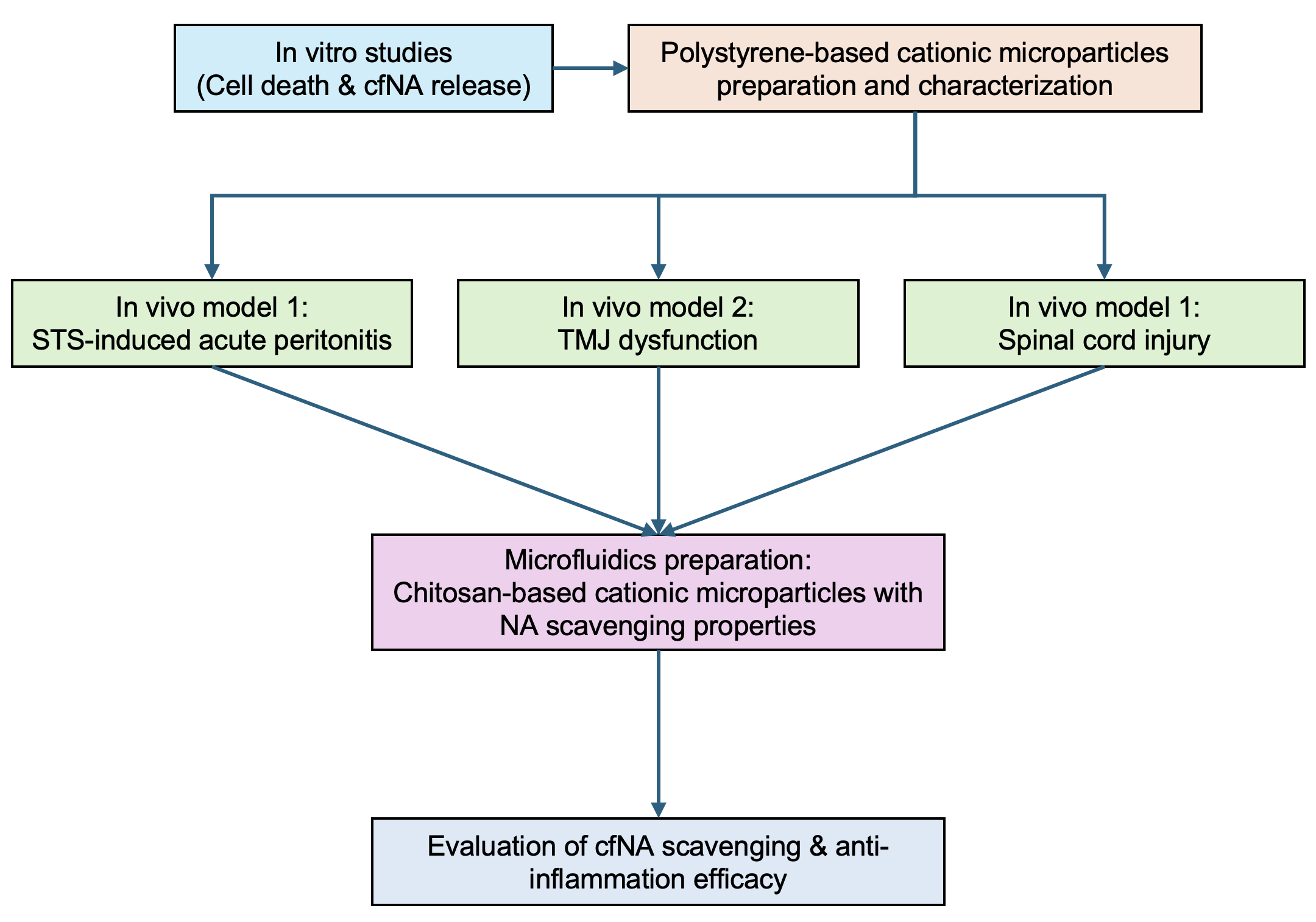
**

**Figure S1. Flow chart of the whole study.**

**Figure S2. Zeta potential of microparticles coated with different concentration of protamine sulfate.**

**
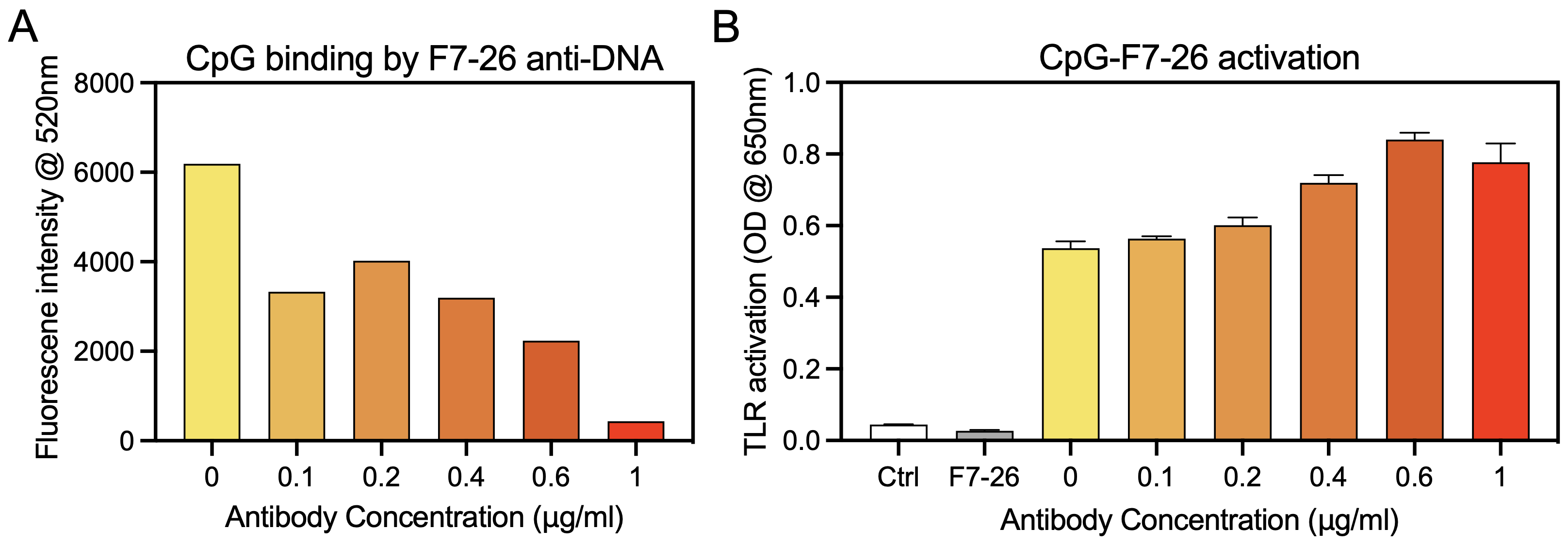
**

**Figure S3. Anti-DNA antibody enhanced pro-inflammatory properties of CpG DNA.** (A) Complexation of CpG with anti-DNA antibody (F7-26) and (B) activation of TLR by the immune complex.


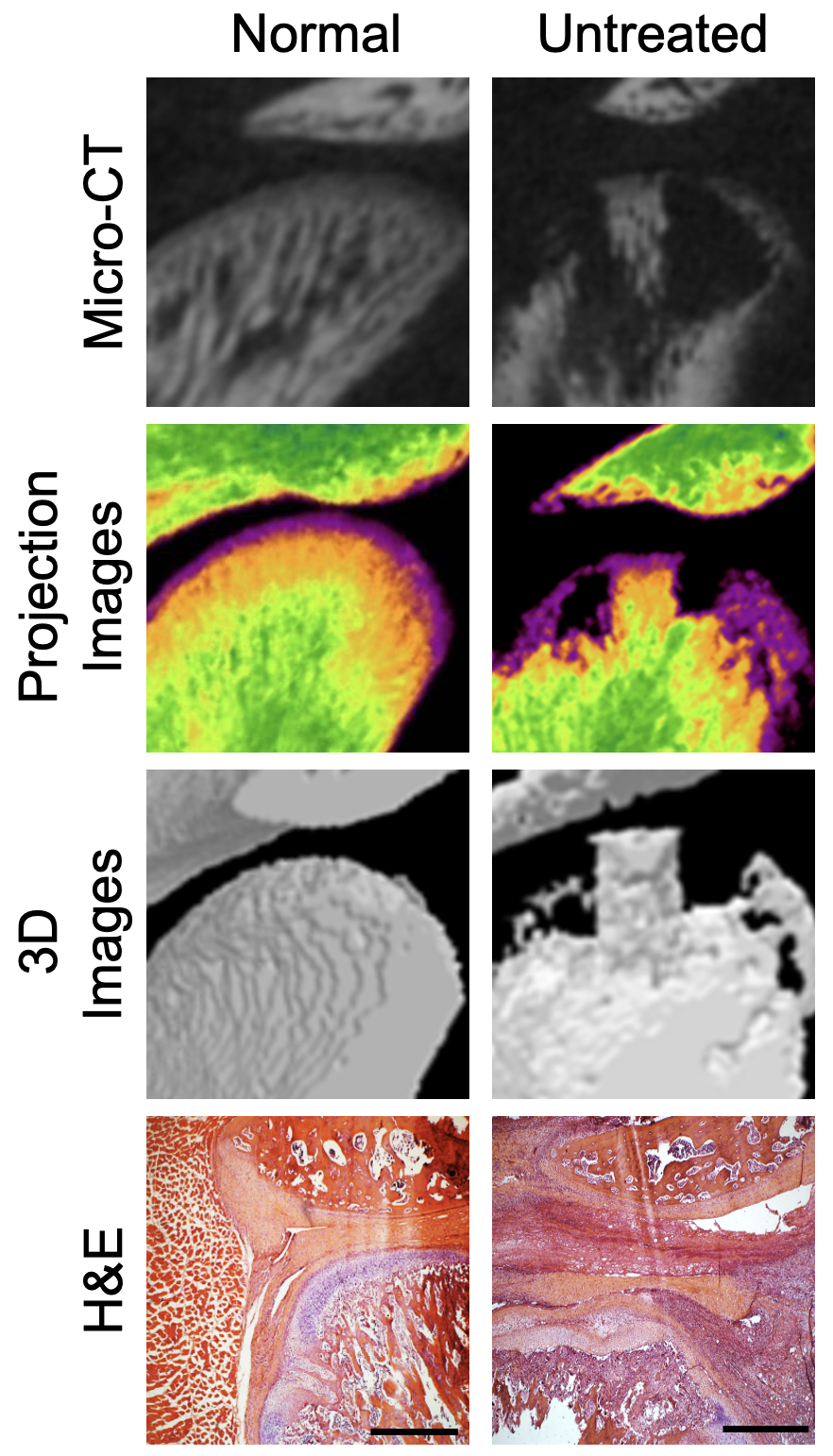


**Figure S4. The TMJ dysfunction mouse model involves persistent inflammation at the TMJ and degradation of the cartilage lining and bone after intraarticular injection of complete Freund’s adjuvant (CFA). Scalebar is 200 μm.**

**
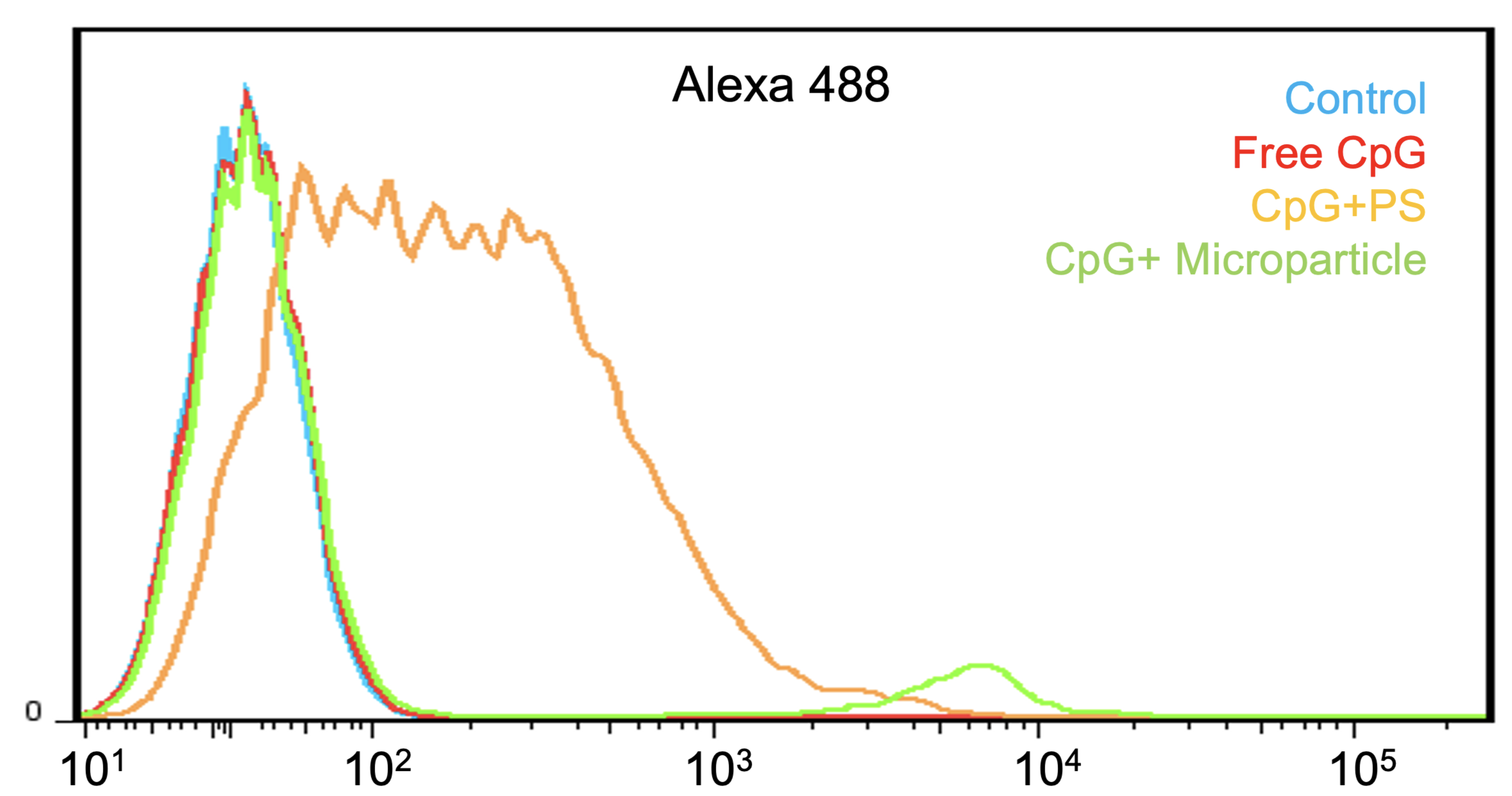
**

**Figure S5. Inhibition of CpG uptake by PS-coated chitosan microparticles in Raw 264.7 cells.**


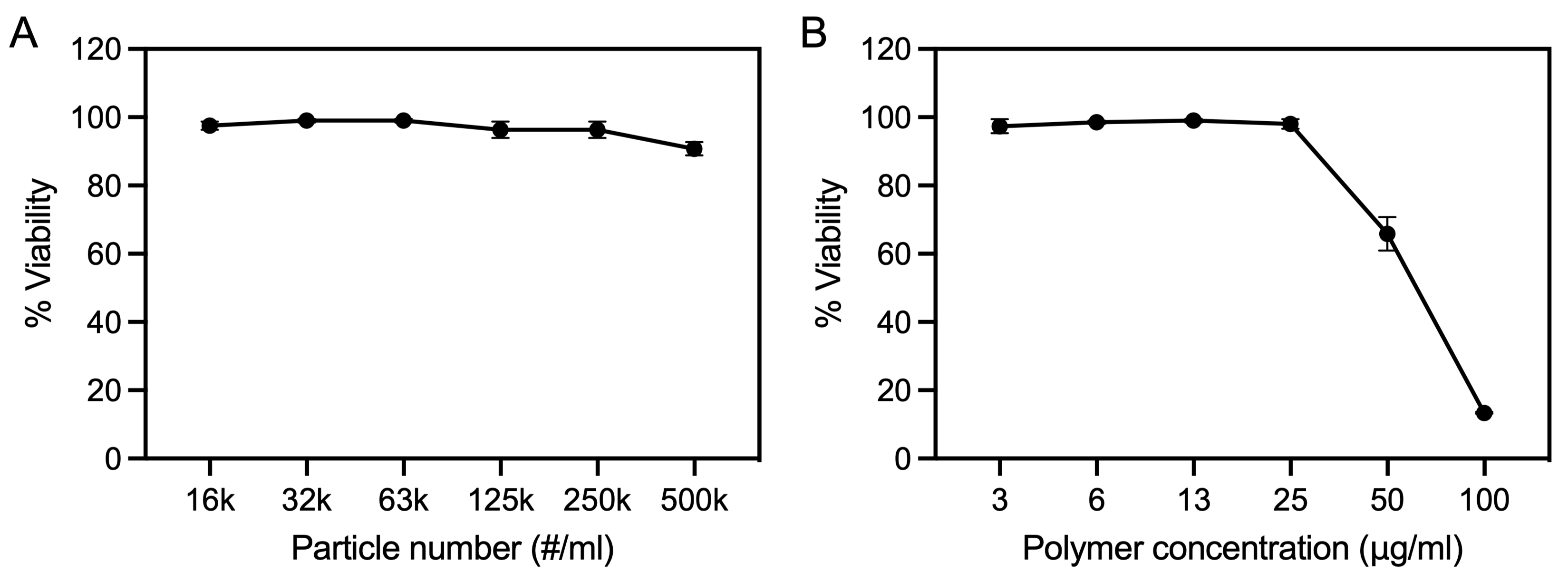


**Figure S6. Cytotoxicity of protamine sulfate-coated chitosan microparticles (A) and free protamine sulfate (B).**
